# Adenosine-Monophosphate-Assisted Homogeneous Silica Coating of Silver Nanoparticles in High Yield

**DOI:** 10.3390/nano13202788

**Published:** 2023-10-18

**Authors:** Carlos Fernández-Lodeiro, Reem Tambosi, Javier Fernández-Lodeiro, Adrián Fernández-Lodeiro, Silvia Nuti, Soufian Ouchane, Nouari Kébaïli, Jorge Pérez-Juste, Isabel Pastoriza-Santos, Carlos Lodeiro

**Affiliations:** 1 Departamento de Química Física, Universidade de Vigo, Campus Universitario Lagoas Marcosende, 36310 Vigo, Spain; carfernandez@uvigo.gal (C.F.-L.); juste@uvigo.es (J.P.-J.); pastoriza@uvigo.es (I.P.-S.); 2 Galicia Sur Health Research Institute (IIS Galicia Sur), SERGAS-UVIGO, 36310 Vigo, Spain; 3 Laboratoire Aimé Cotton (LAC), UMR 9025, Centre National de la Recherche Scientifique (CNRS), Université Paris-Saclay, 91405 Orsay, France; reem.tambosi@universite-paris-saclay.fr; 4 BIOSCOPE Group, LAQV@REQUIMTE, Chemistry Department, Faculty of Science and Technology, University NOVA of Lisbon, Caparica Campus, 2829-516 Caparica, Portugal; a.lodeiro@fct.unl.pt (A.F.-L.); s.nuti@campus.fct.unl.pt (S.N.);; 5 PROTEOMASS Scientific Society, BIOSCOPE Research Group, Departmental Building, Ground Floor, FCT-UNL Caparica Campus, 2829-516 Caparica, Portugal; 6 Institute for Integrative Biology of the Cell (I2BC), UMR 9198, Centre National de la Recherche Scientifique (CNRS), Commissariat à l’Énergie Atomique (CEA), Université Paris-Saclay, 91198 Gif-sur-Yvette, France; soufian.ouchane@i2bc.paris-saclay.fr

**Keywords:** silver nanoparticles, silica coating, mesoporous silica, adenosine monophosphate, bactericidal properties

## Abstract

In this study, we propose a novel approach for the silica coating of silver nanoparticles based on surface modification with adenosine monophosphate (AMP). Upon AMP stabilization, the nanoparticles can be transferred into 2-propanol, promoting the growth of silica on the particle surfaces through the standard Stöber process. The obtained silica shells are uniform and homogeneous, and the method allows a high degree of control over shell thickness while minimizing the presence of uncoated NPs or the negligible presence of core-free silica NPs. In addition, AMP-functionalized AgNPs could be also coated with a mesoporous silica shell using cetyltrimethylammonium chloride (CTAC) as a template. Interestingly, the thickness of the mesoporous silica coating could be tightly adjusted by either the silica precursor concentration or by varying the CTAC concentration while keeping the silica precursor concentration constant. Finally, the influence of the silica coating on the antimicrobial effect of AgNPs was studied on Gram-negative bacteria (*R. gelatinosus* and *E. coli*) and under different bacterial growth conditions, shedding light on their potential applications in different biological environments.

## 1. Introduction

Silver nanoparticles (AgNPs) have gained considerable scientific interest owing to their distinctive optoelectronic and chemical properties. These properties make AgNPs suitable for a wide range of applications, including their use as sensors [1], SERS substrates [2], plasmonic photovoltaic devices [3], antimicrobial agents [4], and even in the medical field [5]. However, the successful integration of Ag nanoparticles into practical applications is hindered by their susceptibility to surrounding chemicals, leading to rapid oxidation and/or aggregation, resulting in substantial degradation of their physicochemical properties. This limitation is especially relevant in the field of antibacterial applications, where the oxidation of AgNPs is desirable. In such scenarios, employing a coating that immobilizes and contains the AgNPs while enabling the controlled release of Ag^+^ can serve as a powerful strategy. Such a coating not only enhances the durability of AgNPs but also facilitates their versatile use as antibacterial materials.

In this scenario, silica (SiO_2_) is an ideal candidate for the external coating of AgNPs. The high stability, controlled porosity, chemical inertness, and optical transparency of silica make it an suitable choice for coating metallic nanoparticles [6,7]. However, despite the numerous advantageous properties of silica, the coating of AgNPs presents challenges due to their susceptibility to oxidation and/or aggregation as well as the limited chemical affinity between silica and the nanoparticle’s surface [6,7].

Different approaches have been proposed to modulate the silica coating of silver NPs via a sol–gel process using silicon alkoxides in an alcoholic medium. Liz-Marzan and coworkers were the first to address this challenge by applying a surface primer, specifically a silane coupling agent, to provide the AgNP surface with silanol anchoring groups and stabilize them in the alcoholic medium [8]. Since then, different polymers or discrete molecules such as PVP [9], arabic gum [10], Daxa19 [11], glucose [12], 2-mercaptoethanesulfonate (MES) [13], or 16-mercaptohexadecanoic acid (MHA) [14] have been proposed to facilitate the controlled deposition of silica on AgNPs [9,10,11,12,13,14]. It should be noted that the direct silica deposition on small citrate-stabilized AgNPs (ca. 10 nm) without additional modifications has been reported by Kobayashi et al. [15]. The authors demonstrated the ability to control the silica thickness with minimal core dissolution using dimethylamine as a catalyst instead of ammonium hydroxide. Although subsequent studies reported silica coating on larger citrate-stabilized AgNPs [16,17,18,19], these synthetic routes often entail certain drawbacks [16,18,20]. 

Although there are several works focused on the dense silica coating of AgNPs, there is a relatively limited body of research on the controlled deposition of mesoporous silica on AgNPs with high yield [21,22,23,24]. For instance, Erten and coworkers and Fathima et al. reported the mesoporous silica coating of AgNPs using cetyltrimethyl ammonium nitrite [22] or dodecyltrimethylammonium bromide [23] as templates, respectively. Although these studies achieved a homogeneous mesoporous silica coating, they did not explore variation in the size of the silver core. On the other hand, other authors reported the direct formation of Ag@mSiO_2_ core@shell nanostructures, where the synthesis of silver AgNPs and the coating with mesoporous silica occur in the same reaction step [24,25]. However, while these direct routes are attractive due to their simplicity, the control of the core size and shell thickness is limited. Consequently, the development of synthetic routes for the mesoporous silica coating of Ag NPs remains highly desirable.

Recently, we reported the dual role played by adenosine monophosphate (AMP) as a shape-directing agent in the synthesis of Au nanostars but also as a robust capping agent for stabilizing the particles in polar organic solvents [26]. Interestingly, the surface modification with AMP conferred vitreophilic properties to the gold surface, thereby facilitating the direct coating of silica onto the nanostars. Based on this previous study, here we study the controlled silica coating of AgNPs assisted by adenosine monophosphate. Furthermore, the ability to achieve either a dense or mesoporous silica coating renders this nanocomposite an ideal platform for studying the impact of the coating on the antimicrobial properties of AgNPs [27,28]. Particularly, we have analyzed the antimicrobial properties of two models of Gram-negative bacteria: *Rubrivivax (R.) gelatinosus and Escherichia (E.) coli.* Different studies have highlighted the detrimental effects on living cells resulting from excessive exposure and/or overuse of heavy metals, including silver. For instance, the toxicity of metals could damage the chloroplasts in plants and algae, impairing the functionality of photosystems. Consequently, these effects can disrupt metabolic processes and hinder the growth of bacterial cells [29,30,31,32]. Since the toxicity mechanisms are not well known, exploring the effect of nanoparticles on the growth of a photosynthetic bacterium such as *R. gelatinosus* can expand the knowledge providing new comprehension regarding the toxic mechanisms in phototrophs. The *R. gelatinosus* is a purple photosynthetic non-sulfur bacteria that can grow by respiration or photosynthesis [33,34,35]. In previous work, *R. gelatinosus* was exposed to silver (Ag^+^) and copper (Cu^+^) ions to identify their target proteins within the respiration and photosynthesis complexes in the cell membrane. That has shown that AgNO_3_ and CuSO_4_ specifically target bacteriochlorophyll, which absorbs light in the near-infrared range at 800 nm (B800), within the light-harvesting complex II (LH2) of the photosystem. These compounds have an impact on the respiratory chain complexes and cause damage to the succinate dehydrogenase (SDH) protein within the first hour of exposure [36]. That study has also shown, in the case of *E. coli*, that Ag^+^ ions have an impact on the succinate dehydrogenase (SDH) and decrease its activity through a longer time of incubation [36]. Here, we have continued our investigation using the same model of bacteria under the stress of different AgNPs; AMP-modified NPs (Ag@AMP), AMP-promoted Ag@SiO_2_ NPs and AMP-promoted mesoporous silica-coated AgNPs (Ag@mSiO_2_ NPs). The in vivo results show the impact of nanoparticles on the cells’ growth under different growth conditions: solid and liquid growth media cultures. Moreover, we exposed bacterial cells to increased concentrations of NPs to detect the toxic point of their reaction, defense mechanism, and methods followed for metal tolerance.

## 2. Materials and Methods

### 2.1. Materials

Silver(I) nitrate (AgNO_3_, 99.9%), trisodium citrate dihydrate (C_6_H_5_Na_3_O_7_·2H_2_O, ≥99.5%), adenosine monophosphate (99%), tetraethyl orthosilicate (99%) and sodium hydroxide (98%) were obtained from Merck KGaA, Darmstadt, Germany. Tannic acid (98%), was obtained from Alfa-Aesar, Ward Hill, MA, USA. Cetyltrimethylammonium chloride (95%) was obtained from TCI Europe NV, Zwijndrecht, Belgium. All reagents were used as received without further purification. Ultrapure water (type I) was used for the preparation of all the water-based solutions. The glassware was cleaned with aqua regia prior to the experiments.

### 2.2. Methods

#### 2.2.1. Synthesis of AgNPs

A 200 mL volume of an aqueous solution containing tannic acid (TA, 0.1 mM) and sodium citrate (SC, 5 mM) was placed in a two-neck round-bottomed flask adapted with a condenser. The solution was heated under vigorous stirring and was maintained for 15 min on boiling. Then 2 mL of AgNO_3_ (25 mM) was quickly injected. The reaction was elapsed by 45 min on boiling and then the solution was cooled under stirring. Then, 100 mL of this colloid solution was purified by centrifugation (8500 rpm for 30 min) to remove the TA/SC excess and redispersed in 39 mL of water reaching [AgNPs]~5.8 × 10^11^ NPs/mL. The concentration of AgNPs expressed as NPs/mL was calculated considering a quantitative reduction in silver and the average size of the AgNPs obtained in the microscopy studies. AgNPs with a mean size of ca. 55 nm were obtained using AgNPs (28 nm) as seeds and growing in 3 rounds in the identical conditions previously reported by N. Bastus et al. [37]. 

#### 2.2.2. Functionalization of AgNPs with AMP

AgNPs were synthesized as described above. Then, 100 mL of obtained AgNPs were centrifugated at 8500 rpm for 30 min and redispersed in 90 mL of SC 2.2 mM. The solution was transferred to a bottom flask and 10 mL of an aqueous solution of AMP (50 mM) was dropped into the solution under vigorous magnetic stirring. The solution was ultrasonicated for 3 min and then stirred for 48 h at room temperature. Finally, the AgNPs were centrifugated at 9000 rpm for 30 min to remove the AMP excess and redispersed in 39 mL of water reaching [AgNPs]~5.8 × 10^11^ NPs/mL. The AgNPs used in mesoporous silica coating were redispersed in 39 mL of NaOH 2 mM instead of water. Larger NPs were functionalized in the same conditions, followed by centrifugation at 6500 rpm for 30 min and redispersed finally in 20.5 mL of water or NaOH 2 mM to reach [AgNPs]~5.8 × 10^11^ NPs/mL. The colloid solutions were stored at 4 °C for further use and were applied as seeds with aging periods ranging from one day to two months.

#### 2.2.3. Dense Silica Coating of AgNPs

A volume of 22.2 mL of isopropanol was placed in a round-bottom flask. Then 5 mL of AgNPs in water ([AgNPs]~5.8 × 10^11^ NPs/mL) was dropped under vigorous stirring. Then, 1.8 mL of DMA was injected into the reaction. After 2 min, 1 mL of iPr solution containing an appropriate amount of TEOS was added under vigorous stirring. The round-bottom flask was capped and maintained under moderate stirring for 24 h at room temperature. Finally, Ag@SiO_2_ NPs were purified by repeated washing by centrifugation at 9500 rpm for 30 min using EtOH as a solvent and finally redispersed in EtOH or water. Coating of larger AgNPs was obtained under the same synthetic strategy. 

#### 2.2.4. Mesoporous Silica Coating of AgNPs

A volume of 24.32 mL of water and 480 μL of CTAC 50 mM were placed in a round-bottom flask. Then 5 mL of AgNPs ([AgNPs]~5.8 × 10^11^ NPs/mL) in NaOH 2 mM was dropped in the reaction under vigorous magnetic stirring. After 5 min, 200 μL of NaOH 0.1 M was injected and the solution was moderately stirred for 24 h. Finally, 1 mL of EtOH with the appropriate quantity of TEOS (see main text) was sequentially dripped (200 μL every 10 min) under stirring. The bottom flask was capped and elapsed for 24 h under moderate stirring at room temperature. The effect of [CTAC] was investigated by increasing the volume of CTAC used (between 480 μL and 900 μL) with the other parameters constants. (Note: [CTAC] and [TEOS] discussed in the results and discussion section were calculated for a total volume of 30 mL, without considering the 1 mL of alcoholic TEOS solution added). Finally, the Ag@mSiO_2_ NPs were purified by 3 rounds of centrifugation 9500 rpm for 30 min in EtOH for electron microscopy analysis. To substantially remove the ammonium template, the NPs were extra purified with 3 additional rounds of centrifugation 9500 rpm for 30 min in MeOH and finally 2 cycles in water. The colloid solutions were ultrasonicated for 3 min between each centrifugation round. Coating of larger AgNPs was obtained under the same synthetic strategy. 

#### 2.2.5. Bacterial Strains and Growth

*E. coli* has grown aerobically (500 mL flasks containing 50 mL medium) at 37 °C in LB medium. *R. gelatinosus* has grown at 30 °C, in the Dark micro-aerobically (low oxygenation: 50 mL flasks containing 50 mL medium) or in light by photosynthesis (filled tubes with residual oxygen in the medium) in malate growth medium. Growth inhibition curves were monitored at OD 680 nm (for *R. gelatinosus)* with measurements taken every 15 min for 24 h using a Tecan Infinite M200 luminometer (Tecan, Mannerdorf, Switzerland) for aerobic conditions. For photosynthesis conditions, strains were grown as described above and OD was measured after 24 h using the Tecan luminometer.

#### 2.2.6. Growth Inhibition Zone on Petri Dishes

For spot inhibition assay, 200 µL (1 OD of cells) of overnight grown cells was mixed with 7 mL semi-solid agar and uniformly spread onto solidified agar plates. After solidification, 5 μL of solutions of AgNPs were spotted on the agar, and then plates were incubated overnight at 37 °C under either photosynthetic for *R. gelatinosus* or respiratory condition for *E. coli.*

#### 2.2.7. Characterizations

The extinction spectra were recorded using a JASCO 770 UV−Vis-NIR spectrophotometer provided by the PROTEOMASS-BIOSCOPE facility (Caparica, Portugal). All spectra were recorded using a HELMA 1 cm light path quartz cell. ζ-potential analysis was carried out in a Malvern ZS instrument at 22 °C in standard 1 mL polystyrene cuvettes provided by the PROTEOMASS-BIOSCOPE facility (Caparica, Portugal). Low-resolution (LR) transmission electron microscopy (TEM) images were obtained using a JEOL JEM 1010 TEM microscope, operating at 100 kV. A JEOL JEM 2010F field-emission gun TEM (JEOL Corporation, Akishima, Tokio, Japan) operating at 200 kV was used to obtain HAADF-STEM images provided by the Centro de Apoio Científico-Tecnolóxico á Investigación, CACTI-UVigo. Particle size, mean size distribution, and silica thickness were calculated from TEM micrographs measuring between 150 and 200 NPs for each sample using the ImageJ package. To prepare samples, 10 μL of colloid solution were drop cast onto a Formvar-coated 400-mesh copper grid (Ted-pella, Inc., Redding, CA, USA) and dried in air. To purify colloid solutions, 15 mL disposable conical tubes were used in an MPW centrifuge. For colloidal stability analysis in alcohol (iPr or EtOH), 1 mL of purified colloid in water (Ag@AT-SC or Ag@AMP NPs) was centrifuged, and the pellet was resuspended in 1 mL of alcohol. This alcoholic solution was then diluted in the corresponding alcohol to obtain spectra. Oxidation studies were performed in a 3 mL quartz cuvette. Ag colloids stored at 4 °C were diluted in water (600 μL of colloid solution in total volume of 3 mL). Subsequently, 200 μL of HCl (0.3 or 0.4 M) were injected into the cuvette, and spectra were recorded every 3 min. After the HCl injection, the cuvette was manually shaken and then left undisturbed during the analysis.

#### 2.2.8. ICP Analysis

Samples of silver nanoparticle concentration used in bacteriological analysis were performed by Icap Q from Thermo Scientific, Waltham, MA, USA. NPs were prepared in H_2_O and measurements are shown in the Table 1.

## 3. Results and Discussion

### 3.1. Synthesis and Characterization of Nanoparticles

First, highly monodisperse AgNPs were synthesized following a previously reported approach in high yield via the reduction in silver nitrate with tannic acid-sodium citrate (TA-SC) developed by N. Bastus et al. [37] with minor modifications, see the experimental section for details. This strategy allows the synthesis of AgNPs in a wide range of sizes with a relatively high monodispersity. As a starting point, we have chosen AgNPs with a mean diameter of 27.9 ± 2.7 nm (denoted Ag@TA-SC NPs), characterized by a well-defined Localized Surface Plasmon Resonance (LSPR) band centered at ca. 406 nm (see Figure S1 in the Supporting Information). Interestingly, the Ag@TA-SC NPs showed good colloidal stability in iPr and EtOH, the two most common organic solvents used for silica-coated AgNPs. The LSPR bands were redshifted to 412 and 411 nm, respectively, due to changes in the dielectric constant of the solvent, but without signals of aggregation (see Figure S2A). This behavior was opposite to that observed in simple citrate-stabilized AgNPs, in which a convenient reduction in the concentration of salts in the colloid solution [17] or the application of silane primer [8] was found to be crucial for transferring the NPs to the alcoholic medium. We selected isopropanol (iPr) as a solvent due to a lower hydrolysis rate of TEOS when compared with EtOH [38] which should minimize the nucleation of core-free silica NPs [12].

Subsequently, the washed AgNPs were coated with silica through a modified Stöber approach using dimethylamine (DMA) as a catalyst based on the limited damping observed in the characteristic LSPR band of citrate-stabilized AgNPs treated with DMA, when compared with ammonia. In addition, more reproducible results are expected due to the lower vapor pressure when compared with ammonia, which limits rapid concentration change [12]. After the injection of DMA, the iPr/water colloid solution did not show destabilization of the AgNPs and although some oxidation was observed after aging, the NPs remained stable even after 24 h (see Figure S2B). Under these conditions, the nanoparticles were successfully coated with silica after the addition of an iPr solution containing TEOS. For instance, using 5 mL of [AgNPs]~5.8 × 10^11^ NPs/mL and [TEOS] = 0.4 mM evolved in a silica coating with negligible presence of uncoated AgNPs. Even so, the surface coating showed an irregular thickness between ~5 and 25 nm. Furthermore, as revealed in the TEM images, a fraction of multicore NPs (~5%) was obtained as a by-product (Figure S3). When [TEOS] was increased to 0.7 mM the mean thickness of the silica coating increased but with a similar multicore NPs subpopulation and the presence of core-free silica nanostructures (Figure S4). Therefore, although Ag@TA-SC NPs without further modifications are stable in iPr, under our conditions, irregular coating formation and a certain percentage of multicore NPs or core-free silica nanostructures can lead to disadvantages in some applications.

To the best of our knowledge, the silica coating of Ag@TA-SC NPs without any additional modification was not reported before. Next, to avoid the nucleation of free silica, an irregular silica coating, and the formation of multicore NPs we explore the ability of AMP to assist the silica coating. Thus, Ag@TA-SC NPs were functionalized with AMP via incubation (see experimental section for details). The surface modification of the nanoparticles (hereinafter denoted as Ag@AMP NPs for clarity) does not modify the optical properties of the NPs. The presence of AMP on the AgNPs surface was confirmed by surface-enhanced Raman scattering (SERS) spectroscopy showing an intense signal at 731 cm^−1^ assigned to the ring breathing mode of AMP molecules (see Figures S5 and S6) [39]. In addition, the SERS characterization shows the presence of additional bands indicating that tannic acid and citrate ions remain on the nanoparticle surface. In terms of colloidal stability, Ag@AMP NPs exhibited similar solubility in an alcoholic medium and increased stability upon contact with DMA (Figure S7). However, comparative oxidation studies of Ag@AT-SC and Ag@AMP NPs with HCl reveal the cooperative effect of AMP to protect the Ag surface against oxidation (Figure S8).

**Figure 1:**
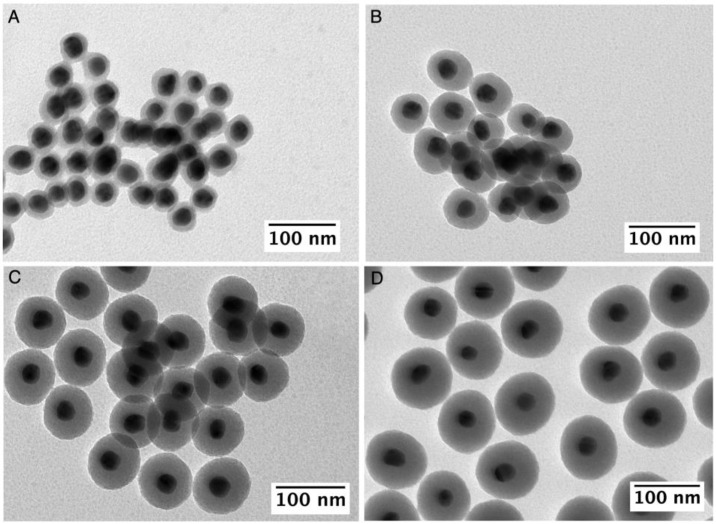
TEM images of Ag@SiO_2_ NPs synthesized using 5 mL of [AgNPs]~5.8 × 10^11^ NPs/mL as seeds and with [TEOS] of 0.37 (**A**), 0.67 (**B**), 0.90 (**C**), and 1.5 mM (**D**).

Figure 1 shows representative TEM images of the Ag@AMP NPs coated with homogenous silica shells obtained by increasing the [TEOS] concentration from 0.37 to 1.5 mM while keeping the Ag@AMP NPs concentration constant. Interestingly, the surface modification with AMP allowed the control of the silica shell thickness between ca. 10 and 35 nm and, more importantly, without uncoated AgNPs, negligible formation of multicore or core-free NPs (see Figures S9–S13). The optical properties of the obtained Ag@SiO_2_ NPs show a redshift in the position of the LSPR band compared to the uncoated NPs due to the increase in the local refractive index surrounding the particles (see Figures S9A–S13A in the SI) [40]. Notably, previous works have explored the formation of a silica shell over silver nanoparticles (NPs) by manipulating factors such as pH, EtOH/water ratio, or concentration of silica precursor between other parameters [8,15,19]. In our study, we held the remaining variables constant while varying only the [TEOS]. This approach allowed us to focus our analysis on assessing the volume fraction of silica within the core@shell nanoparticles according to the quantity of silica precursor used. Our results reveal a distinct linear correlation between the extent of silica growth (quantified by the volume of formed silica) and the employed TEOS concentration (see Figure S14). This linear relationship strongly indicates a controlled and predictable silica growth process within the silver nuclei, seemingly independent of any noticeable contribution from auto-nucleation silica processes. These findings align with observations derived from TEM analysis.

Interestingly, the AMP-assisted silica coating procedure could be extended to larger AgNPs (mean size ca. 55 nm), as shown in Figures S15 and S16, where the thickness of a homogeneous silica coating can be easily tuned by adjusting the TEOS concentration. 

Next, we explored the mesoporous silica coating of Ag@AMP NPs mediated by quaternary ammonium surfactants as template agents. Particularly, we have chosen CTAC instead of CTAB because the Stöber process can be performed at room temperature while CTAB tends to crystalize at temperatures below 25 °C [41]. Considering previous research, it has been observed that exceeding the critical micellar concentration (CMC) when using surfactants as template agents for silica coating of metal nanoparticles can lead to uneven coating or even a lack of silica deposition on the metal nanoparticles [42,43]. In our case, we are deliberately keeping the CTAC concentration below the reported CMC ca. 1.1 mM [44,45]. This approach aims to reduce micelle formation in the solution, promoting a more controlled and effective coating.

**Figure 2:**
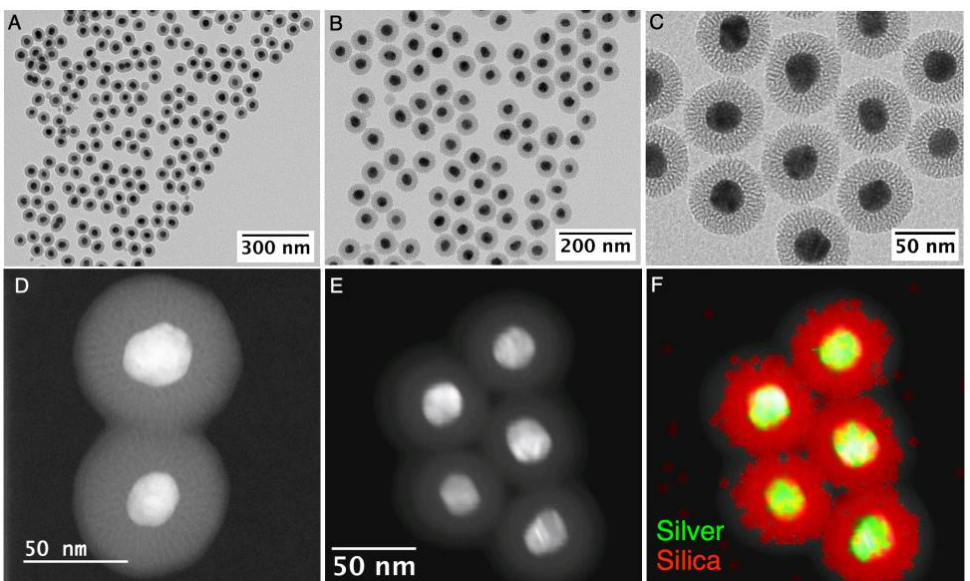
TEM (**A**–**C**) and HAADF-STEM (**D**,**E**) images, at different magnifications, of Ag@mSiO_2_ synthesized using 5 mL of [AgNPs]~5.8 × 10^11^ NPs/mL with [CTAC] and [TEOS] of 0.8 and 3 mM, respectively In (**F**), the EDS elemental mapping of image (**E**) is presented, with the overlay of Ag L (green) and Si K (red).

The stability of Ag@AMP NPs upon redispersion in CTAC/NaOH solutions is not compromised as demonstrated by the negligible damping of the LSPR band and without changes in λ_max_ after 24 h, even in the presence of sodium hydroxide to increase the pH in a range of 10.5–11 (see Figure S17). Subsequently, the addition of an ethanolic solution of TEOS (see experimental section) led to AgNPs coated with a highly homogeneous mesoporous silica shell. As shown in Figure 2A,B, using 5 mL of [AgNPs]~5.8 × 10^11^ NPs/mL and with [CTAC] and [TEOS] of 0.8 and 3 mM, respectively; a highly defined and homogeneous mesoporous silica shell with an average thickness of 18.9 ± 1.9 nm was formed in high yield over AgNPs. TEM and HAADF-STEM images revealed that silica presents channels that cross the shell from the silver core to the outside (Figure 2C,D) similar to that observed in other metal nanoparticles coated with silica using DTAB or CTAB as templates [23,43]. The formation of Ag@mSiO_2_ core@shell nanostructures was confirmed by energy-dispersive X-ray spectroscopy (EDS) combined with STEM. The elemental map obtained showing the relative distribution of the silver in the center and silica in the shell confirms the composition and core@shell nanostructure (see Figure 2E,F and Figure S18).

**Figure 3:**
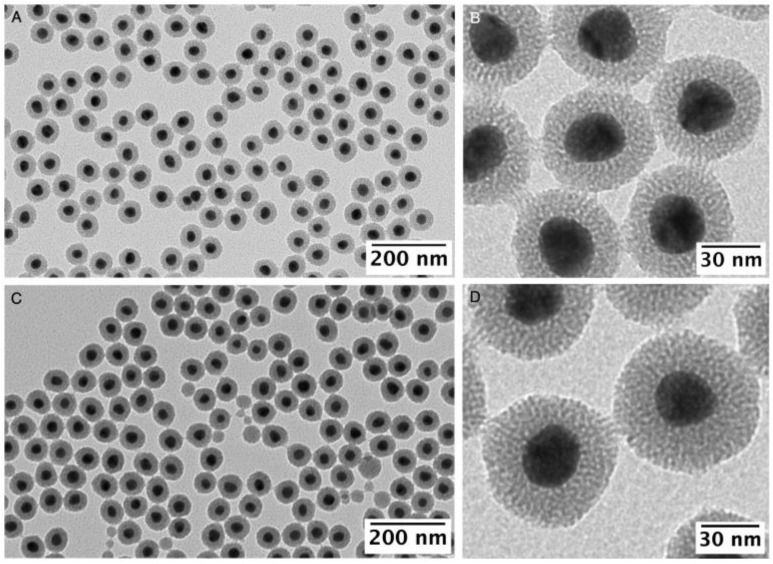
TEM images of Ag@mSiO_2_ synthesized using 5 mL of [AgNPs]~5.8 × 10^11^ NPs/mL, [CTAC] = 0.8 mM and with [TEOS] of 2.24 mM (**A**,**B**) and 3.75 mM (**C**,**D**) ranging silica thickness of 15.9 ± 1.4 nm and 22.1 ± 2.2 nm, respectively.

Control experiments showed that the mesoporous silica thickness can be controlled between 15.9 ± 1.4 and 22.1 ± 2.2 nm when [TEOS] was increased from 2.24 to 3.75 mM (see Figure 2 and Figure 3). At TEOS concentrations below 1.5 mM no silica shell was formed (Figure S19), most probably due to the higher solubility of TEOS in the aqueous surfactant solution. At TEOS concentrations above 4.5 mM a relatively high population of core-free silica NPs is observed, but no significant increase in the thickness of the mesoporous silica shell which remained almost constant at ca. 23 nm in the coexisting core@shell nanoparticles (Figure S20A,B). Our results demonstrate a straightforward link between the amount of mesoporous silica growth (measured by the volume of silica formed) and the concentration of TEOS used within a range of 2.24 and 3.75 mM which produce a high yield of Ag@mSiO_2_ (see Figure S20C).

**Figure 4:**
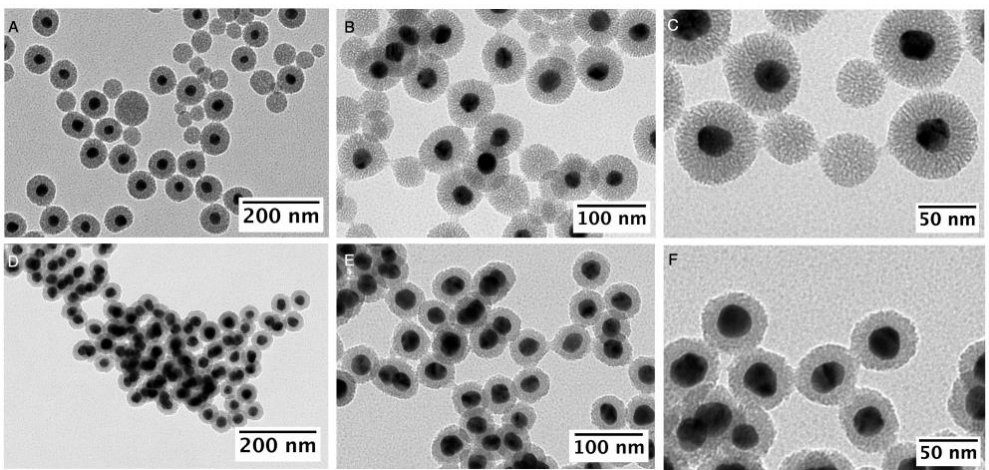
TEM images of Ag@mSiO_2_ obtained using 5 mL of [AgNPs]~5.8 × 10^11^ NPs/mL, [TEOS] of 3 mM and with [CTAC] of 0.6 mM (**A**–**C**) and 1 mM (**D**–**F**).

Regarding the optimal CTAC concentration to promote a homogenous mesoporous silica coating, 0.8 mM was chosen due to the negligible presence of core-free mesoporous SiO_2_ NPs, despite a small fraction of multicore NPs (~1–2%, see Figure 2A–C). Interestingly, at CTAC concentrations below 0.6 mM the NPs the formation of an important subpopulation of core-free SiO_2_ NPs (~30–40%) was observed (Figure 4A–C). Conversely, higher CTAC concentrations (ca. 1 mM) led to a decrease in the silica thickness to ca. 15 nm and without evidence of core-free NPs formation (Figure 4D–F). Our results are in line with a surfactant-dependent regimen, which was previously observed in the silica coating of gold nanorods using CTAB [43,46].

**Figure 5:**
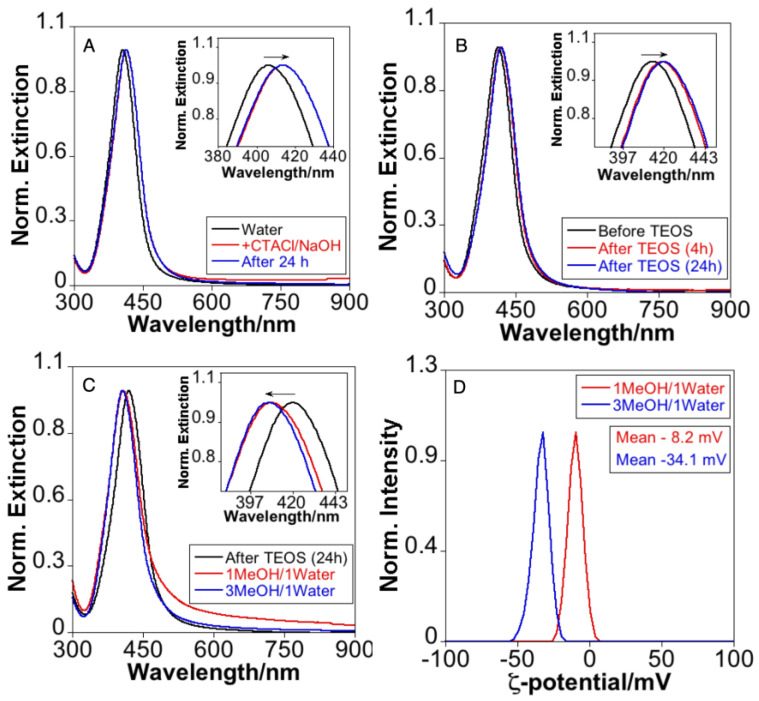
(**A**) Comparative extinction spectra of Ag@AMP NPs in water or CTACl/NaOH. (**B**) Comparative extinction spectra of Ag@AMP NPs before and after TEOS addition. (**C**) Comparative extinction spectra of Ag@mSiO_2_ NPs when different extra-purification cycles in MeOH and water were performed. (**D**) ζ-potential of Ag@mSiO_2_ after different extra-purification cycles.

The optical properties of the mesoporous silica coating procedure were followed by UV-Vis-NIR spectroscopy. As shown in Figure 5A, the LSPR band of Ag NPs was redshifted to ca. 414 nm upon redispersion in the aqueous CTACl/NaOH solution and remained constant for at least 24 h. After the addition of TEOS, the LSPR band showed a gradual redshift to ca. 420 nm (Figure 5B). Interestingly, after repeated purification cycles using MeOH and water, the LSPR band exhibited a blue shift from 420 to 407 nm (Figure 5C). Although partial oxidation of the silver nuclei cannot be ruled out, we attributed this blue shift, to the dissolution of CTA(+) used as a template that remains between the Ag surface and the silica shell and/or in the internal pores of this mesostructured layer. These observations are consistent with those previous results observed in mesoporous silica-coated gold nanorods [28,41,47]. Furthermore, no appreciable changes in the silver core dimension were observed after the inspection of TEM images of Ag@mSiO_2_ purified in MeOH (Figure S21). The ζ-potential shifted to a negative value during purification, obtaining a stable value of −34.1 mV (Figure 5D), which is like that reported for purified Ag@mSiO_2_ NPs obtained using dodecyltrimethylammonium bromide (DTB) as a surfactant [23].

Furthermore, the present mesoporous silica coating strategy can be easily applied to larger Ag NPs by simply adjusting the CTAC and TEOS concentrations (Figure S22). Importantly, unlike PVP-functionalized AgNPs for silica coating, which cannot be stored for long periods (less than two days) [9], AMP-functionalized AgNPs showed similar results with a minimum of two months, when stored at 4 °C (see Figure S23).

### 3.2. Antimicrobial Studies

Given that a highly homogeneous silica coating (dense or mesoporous) can be imparted to AgNPs of different sizes, in the next step we study the influence of the nature and structure of the silica coating on the antimicrobial properties of AgNPs. As a proof of concept, AgNPs with an average diameter of ca. 28 nm were selected. These nanoparticles are not anticipated to penetrate the bacterial cell membrane, thus allowing us to attribute any observed antimicrobial effects primarily to the release of Ag^+^ ions [48]. 

It has been previously observed that the silica coating on AgNPs can attenuate their antimicrobial effect without eliminating it [27]. Therefore, in the present study, we explore the bactericidal potential associated with the different coatings; two dense silica thicknesses (namely 9.3 ± 1.8 nm and 16.1 ± 2.8 nm) and a mesoporous silica coating of 18.9 ± 1.9 nm (see Table 2). As a reference, we also studied the starting uncoated AgNPs (Ag@AT-SC NPs) and the AMP surface modification (Ag@AMP NPs). Particularly, we investigated the antimicrobial effects in the minimum inhibitory concentration (MIC) ranges lower than or like those reported in the literature for both types of bacteria. This approach allowed us to discern any differences between the analyzed colloids rather than focusing on their toxicity at high concentrations.

**Figure 6:**
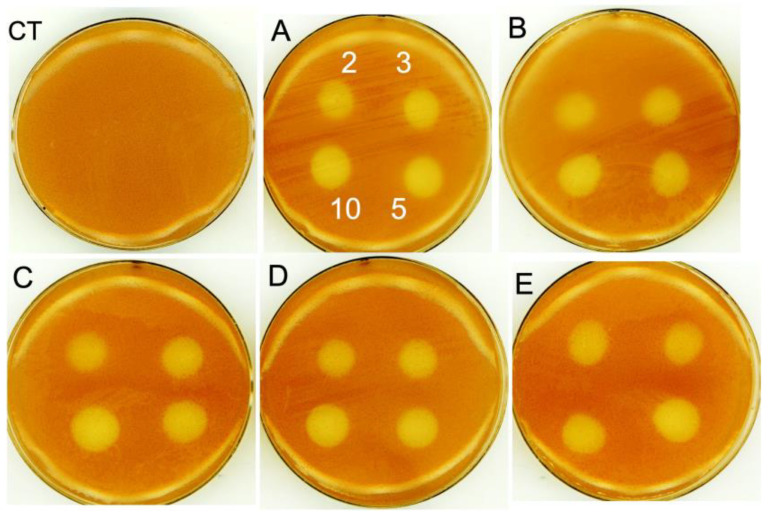
Growth inhibition zones of *R. gelatinosus* grown under photosynthetic conditions on Petri dishes, without (CT) or with AgNPs (samples **A**–**E**) with concentrations ranging from 2 to 10 μg/mL, CT: control without AgNPs addition. The numbers in (**A**) indicate the explored concentration values (μg/mL) and their location, which maintained constant in (**B**–**E**).

First, we exposed *R. gelatinosus* cells grown on solid agar to AgNPs (samples A–E). Petri dishes were prepared with a malate medium in which the bacteria were inoculated (see materials and methods) and drops of increasing concentrations of AgNPs were spotted on the agar. Plates were incubated overnight at 37 °C under light and anaerobic conditions to check the effect of the NPs on photosynthetic growth. Figure 6 shows inhibition zones of growth and the impact on *R. gelatinosus* cells in the presence of 2, 3, 5, and 10 μg/mL of each sample. Obviously, all samples (A–E) of AgNPs have the same effect on growth in the Petri dishes despite their differences in particle coating thicknesses.

In the second experiment, AgNPs were included in the agar medium, and then serial dilutions of bacteria were spotted on the solidified agar plates to assess their ability to deal with AgNPs (Figure S24). In this assay, only concentrated *R. gelatinosus* cells were able to grow in the presence of AgNPs, while no growth was observed when diluted cells were spotted. A high bacterial cell count is indicative of defense mechanisms against environmental and metal stress [49]. Altogether, our data showed that AgNPs inhibit the photosynthetic growth of *R. gelatinosus* regardless of the coating thicknesses. Since the size of the AgNPs prevents them from crossing the cell membrane, it was concluded that the inhibitory effect should arise from Ag^+^ release in the growth medium. 

For *E. coli*, similar experiments were conducted but under aerobic conditions in the LB medium. The results shown in Figures S25 and S26 indicated that in contrast to *R. gelatinosus, E. coli* cells were not affected by AgNPs, nor when spotted on cells on the agar plates (Figure S25), and nor when mixed in culture media (Figure S26).

Given that the growth inhibition assay on a solid medium led to the immobility of the NPs, which can minimize the release of silver through the different coating of NPs, we decided to investigate the growth pattern of *R. gelatinosus* and *E. coli* under the stress of NPs in shacked liquid media. Growth in the absence or presence of AgNPs was followed using a TECAN plate reader only under aerobic conditions.

**Figure 7:**
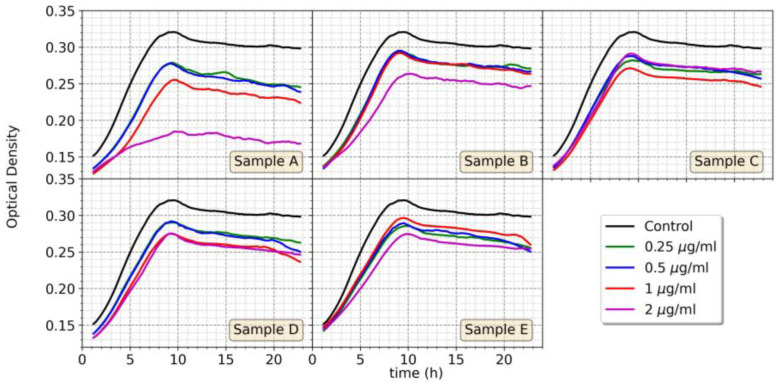
Growth curves of *R. gelatinosus* under aerobic conditions in the absence or presence of AgNPs (**A**–**E**) with concentrations ranging from 0.25 to 2 μg/mL.

The results in Figure 7 show that in general all samples (B to E) with concentrations: 0.25, 0.5, 1, and 2 μg/mL have a toxic effect on the aerobic growth of *R. gelatinosus* causing a delay in the growth and impact the growth rate. Indeed, for all samples and concentrations, the cell density of exposed cells was lower than that of the untreated control culture. Interestingly, sample A (Ag@AT-SC NPs) showed a stronger impact on cell growth as the growth inhibition was more pronounced. The inhibition in the presence of Ag@AT-SC NPs was concentration-dependent and the growth was almost completely inhibited at 2 μg/mL concentration. In comparison with Ag@AT-SC NPs, this result seems to indicate that the functionalization with AMP decreases the toxicity of AgNPs. 

When higher concentrations (3, 5, and 10 μg/mL) of AgNPs were used, *R. gelatinosus* cells were sensitive and unable to tolerate above 2 μg/mL of any NPs as seen in Figure S27. Table 3 summarizes the sensitivity of *R. gelatinosus* to different concentrations of AgNPs.

**Figure 8:**
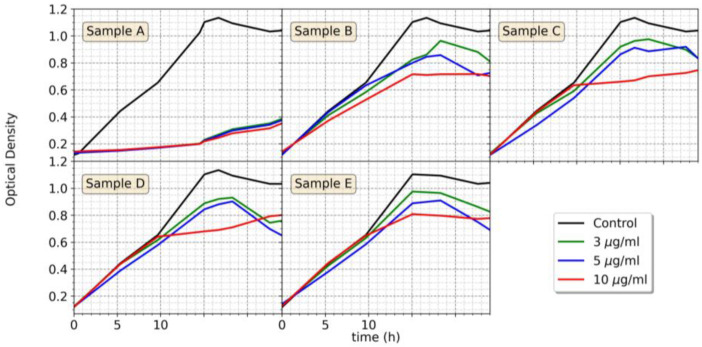
Growth curve of *E. coli* under aerobic conditions in the absence or presence of AgNPs (samples **A**–**E**) with concentrations ranging from 3 to 10 μg/mL.

In the case of *E. coli*, the results shown in Figure S28 indicate that at low concentrations between 0.25 and 2 μg/mL, most AgNPs (samples C–E) had almost no or slight (samples A–B) effect on cell growth. However, when the AgNPs concentration was increased (3, 5, and 10 μg/mL), the *E. coli* growth was affected (Figure 8), in particular, as for *R. gelatinosus*, the Ag@AT-SC NPs (sample A) were more toxic than the other AgNPs (samples B–E) that exhibited only limited inhibition towards *E. coli*. Indeed, while 3 μg/mL of AgNPs@AT-SC (sample A) was efficient in completely inhibiting the growth of *E. coli*, 10 μg/mL of the AMP-functionalized or silica-coated AgNPs (samples B–E) were required to partially inhibit *E. coli* growth. Table S1 summarizes the sensitivity and resistance of *E. coli* to the different AgNP concentrations studied. 

Based on these results, we can conclude that *R. gelatinosus* can tolerate low concentrations (less than 2 μg/mL) of AgNPs compared to *E. coli* which showed higher tolerance both on solid and in liquid media to different AgNPs (samples B–E) tested. Furthermore, our data revealed that the AgNPs@AT-SC NPs (sample A) were more efficient as inhibitors of growth, including *E. coli* in liquid and shacked media, which aligns with the range reported in the literature for other AgNPs [50,51]. Interestingly, after AMP functionalization, the AgNPs showed higher MIC ranges, approaching those obtained for silica-coated NPs. It is worth noting that despite obtaining higher MICs, AgNPs coated with dense or mesoporous silica showed comparable MICs to that informed to other nanomaterials composed of silver/silica nanomaterial [51].

To explain the differences between *E. coli* and *R. gelatinosus* tolerance, we should emphasize the difference in the media chemical composition (malate medium versus LB medium), the difference in component of the cell membrane, and the tolerance ability towards Ag^+^ ions of each strain. Indeed, *R. gelatinosus* is more sensitive to Ag^+^ (1 µM Ag^+^ inhibits growth in malate medium) than *E. coli* in LB medium in which up to 20 µM Ag^+^ is required to inhibit growth. This difference is likely to be due to the chelation ability of Ag^+^ by medium components in LB, but also to the efficiency of the *E. coli* efflux pumps Cop/Cus that expel Ag^+^ from the cells compared to *R. gelatinosus.*

## 4. Conclusions

We have successfully developed a highly efficient method for producing uniform silica coatings on silver nanoparticles. In our approach, adenosine monophosphate (AMP) serves as a co-stabilizer for pre-formed silver nanoparticles, playing a crucial role in facilitating controlled dense silica coating with thicknesses ranging from approximately 10 to 35 nm. Furthermore, we investigated the growth of mesoporous silica shells using the Ag@AMP NPs as seeds and cetyltrimethylammonium chloride (CTAC) as a template. We found that the growth of mesoporous silica shells can be controlled by adjusting either the surfactant (CTAC) or the concentration of the silica precursor (TEOS). We examined the antimicrobial properties of silver nanoparticles with different coatings. Our results showed that the presence of a silica coating, whether dense or mesoporous, did not compromise the antibacterial potential of the nanoparticles, at least up to a thickness of approximately 19 nm. It is worth noting that while we observed a potential antimicrobial effect of the investigated nanoparticles on *R. gelatinosus* at low minimum inhibitory concentrations (MICs) of less than 3 μg/mL, a significant decrease in antimicrobial effect against *E. coli* was observed, even at higher concentrations ranging from 5 to 10 μg/mL. However, Ag@TA-SC NPs demonstrated a strong bactericidal effect against *E. coli* bacteria at concentrations between 3 and 10 μg/mL which manifested the additional protection offered by AMP to the silver surfaces, preventing oxidation. The outer silica layer provides several advantages, such as increased stability, easy secondary functionalization, and a mesostructured nature with potential applications as a molecular carrier, and importantly, it does not impede the release of Ag^+^ ions. Future research efforts should be directed towards finding ways to improve the bacterial properties of silver nanoparticles coated with silica, such as changes in the surface charge, organic functionalization, or drug loading which can provide solutions to develop more durable and safe antimicrobial materials.

## Figures and Tables

**Table 1:** Silver concentrations obtained from the samples analyzed in the bacteriological studies.

Sample	Identification		Size Core/Shell (nm)	Concentration (μg/mL)
A	Ag@TA-SC	NPs	28/-	26.58
B	Ag@AMP	NPs	28/-	50.02
C	Ag@SiO_2_	NPs	28/10	63.36
D	Ag@SiO_2_	NPs	28/16	54.12
E	Ag@mSiO_2_	NPs	28/19	59.92

**Table 2:** List of the different colloids tested in the antimicrobial studies (the silver core size showed were determined from the TEM images of the final core@shell NPs utilized in the study).

Sample	Identification	Core Size/nm	Shell Thickness/nm	Type of Si
A	Ag@AT-SC NPs	28.3 ± 2.6	-	-
B	Ag@AMP NPs	28.8 ± 2.5	-	-
C	Ag@SiO_2_ NPs	28.4 ± 2.8	9.3 ± 1.8	Dense
D	Ag@SiO_2_ NPs	28.5 ± 2.6	16.1 ± 2.8	Dense
E	Ag@mSiO_2_ NPs	28.9 ± 2.8	18.9 ± 1.9	Mesoporous

**Table 3:** Minimal inhibitory concentration (MIC) value for *R.gelatinosus* growth pattern.

AgNPs	MIC (µg/mL)	Interpretation in Solid Medium	MIC (µg/mL)	Interpretation in Solution Medium
A	<2	Sensitive	≤2	Sensitive
B	<2	Sensitive	<3	Sensitive
C	<2	Sensitive	<3	Sensitive
D	<2	Sensitive	<3	Sensitive
E	<2	Sensitive	<3	Sensitive

## Data Availability

The data that support the findings of this study are available from the corresponding authors upon request.

## Supplementary Materials

The Supporting Information can be downloaded at: https://www.mdpi.com/article/10.3390/nano13202788/s1. References [52,53] are cited in the Supplementary Materials.
