# Supplementary material for: Adenosine-Monophosphate-Assisted Homogeneous Silica Coating of Silver Nanoparticles in High Yield
Source: Nanomaterials (Basel). 2023 Oct 18;13(20):2788. doi: 10.3390/nano13202788 (PMC10609066; doi:10.3390/nano13202788)
Supplement: Supplementary file 1 [file nanomaterials-13-02788-s001.zip › nanomaterials-2652685-supplementary.pdf]

Adenosine-Monophosphate-Assisted Homogeneous Silica Coating of Silver Nanoparticles in High Yield

Carlos Fernández-Lodeiro [1,2], Reem Tambosi [3], Javier Fernández-Lodeiro [4,5,*], Adrián Fernández-Lodeiro [4,5], Silvia Nuti [4,5], Soufian Ouchane [6], Nouari Kébaïli [3,*], Jorge Pérez-Juste [1,2], Isabel Pastoriza-Santos [1,2] and Carlos Lodeiro [4,5]

1 Departamento de Química Física, Universidade de Vigo, Campus Universitario Lagoas Marcosende, 36310 Vigo, Spain; carfernandez@uvigo.gal (C.F.-L.); juste@uvigo.es (J.P.-J.); pastoriza@uvigo.es (I.P.-S.)

2 Galicia Sur Health Research Institute (IIS Galicia Sur), SERGAS-UVIGO, 36310 Vigo, Spain

3 Laboratoire Aimé Cotton (LAC), UMR 9025, Centre National de la Recherche Scientifique (CNRS), Université Paris-Saclay, 91405 Orsay, France; reem.tambosi@universite-paris-saclay.fr

4 BIOSCOPE Group, LAQV@REQUIMTE, Chemistry Department, Faculty of Science and Technology, University NOVA of Lisbon, Caparica Campus, 2829-516 Caparica, Portugal; a.lodeiro@fct.unl.pt (A.F.-L.); s.nuti@campus.fct.unl.pt (S.N.); cle@fct.unl.pt (C.L.)

5 PROTEOMASS Scientific Society, BIOSCOPE Research Group, Departmental Building, Ground Floor, FCT-UNL Caparica Campus, 2829-516 Caparica, Portugal

6 Institute for Integrative Biology of the Cell (I2BC), UMR 9198, Centre National de la Recherche Scientifique (CNRS), Commissariat à l'Énergie Atomique (CEA), Université Paris-Saclay, 91198 Gif-sur-Yvette, France; soufian.ouchane@i2bc.paris-saclay.fr

* Correspondence: j.lodeiro@fct.unl.pt (J.F.-L.); nouari.kebaili@universite-paris-saclay.fr (N.K.)

Supporting Information – (Figures S1 to S28 and Table S1)

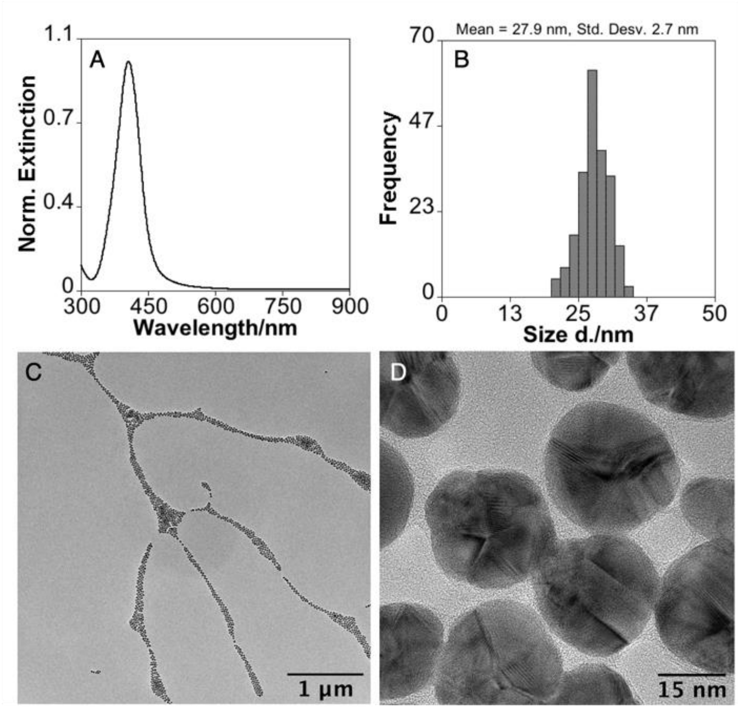

Figure S1. Normalized extinction spectra (A), histogram (B), and TEM images (C) of obtained AgNPs obtained.

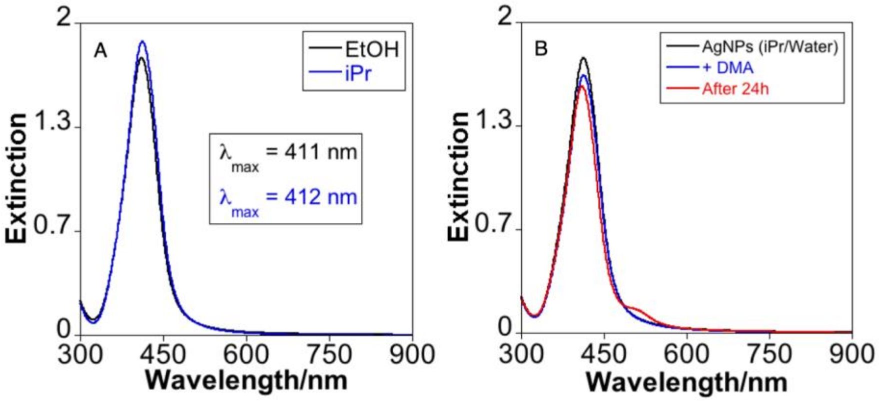

Figure S2. Normalized extinction of Ag@AT-SC NPs in EtOH and iPr (A). Ag@AT-SC NPs in iPr/Water after injection of DMA and their ageing for 24h (B).

The plasmon damping in AgNPs is contingent upon the chemical nature of the nanoparticle interface [1]. This effect, noted after DMA interaction, has been previously studied in citrate-stabilized AgNPs and compared with other amines like ammonia and methylamine (MA) [2]. For instance, ammonia led

to a rapid damping of the surface plasmon band over time, reducing the intensity at the maximum to approximately half of the initial value. The observed change in the λ_{max} of the LSPR band, coupled with the associated plasmon damping, is attributed to the aerial oxidation of Ag(0) in the presence of ammonia. This process results in the dissolution of Ag(0) in water, forming $Ag(NH_3)_2^+$ complex ions [2]. Conversely, using methylamine or dimethylamine resulted in a minimal decrease in extinction intensity at the maximum, indicating that the optical effect is related to amine adsorption on the surface rather than Ag(0) oxidation.

In our case, in the AgNPs stabilized with AT-SC, a similar effect was observed after interaction with DMA (Figure S2A). Notably, prolonged contact not only intensified the damping but also blue-shifted the λ_{max} of the LSPR band (Figure S2B), unequivocally indicating some level of oxidation of the metal cores.

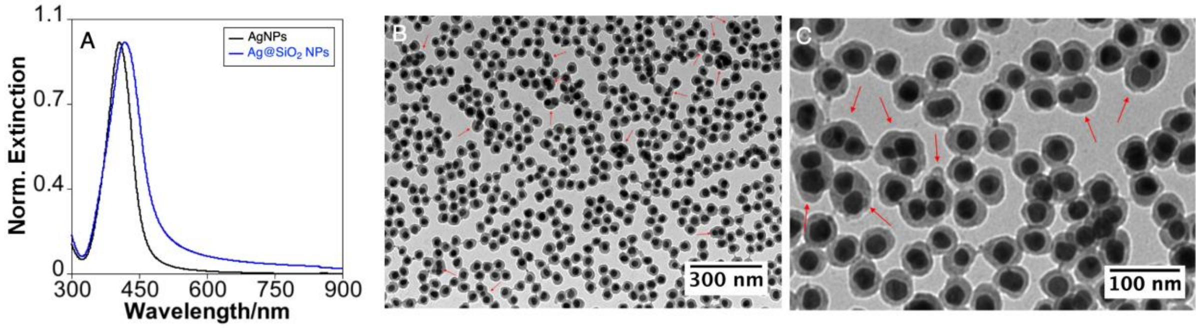

Figure S3. Normalized extinction spectra (A) and TEM images (B) of $Ag@SiO_2$ NPs obtained using 5 mL of [Ag@AT-SC]~$5.8x10^{11}$ NPs/mL as seeds and [TEOS]=0.4 mM. Red arrows in B and C shown the multicore NPs.

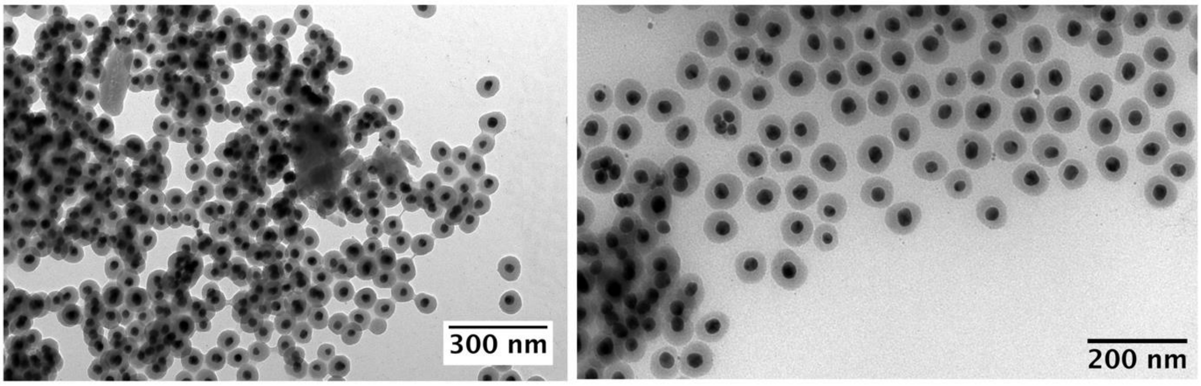

Figure S4. TEM images of Ag@Si NPs obtained using 5 mL of [Ag@AT-SC]~5.8×10^{11} NPs/mL as seeds and [TEOS]= 0.7 mM.

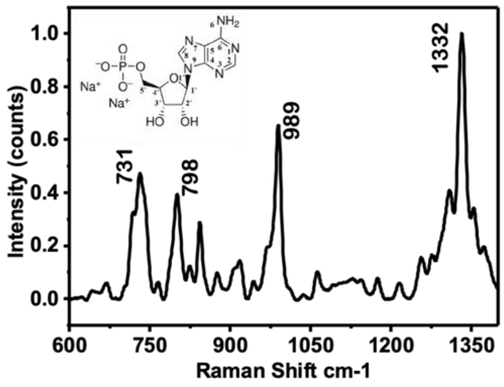

SERS peak position (cm^{-1})	Peak assignment
731	Ring breathing
798	πHC8N9N7
989	$\nu_s PO_3^{2-}$
1332	ν ring (5)

Figure S5. RAMAN spectra obtained for AMP (inset show the molecular structure). The table show the assignation of the more intense signals in the AMP spectra.

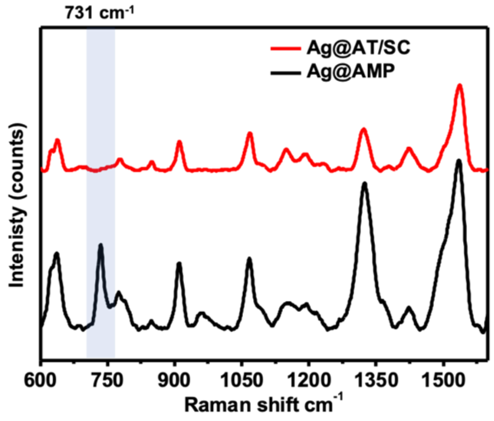

Figure S6. Comparative SERS spectra obtained for Ag@AT-SC and Ag@AMP NPs.

The Ag@AMP NPs were centrifugated and resuspended in water 2 times before SERS studies. It can be seen in the comparative spectra that in addition to presenting signals assignable to AT and SC, the functionalized Ag@AMP presented an intense additional band centered on *ca.* 731 cm^{-1}, originated by the ring breathing of the AMP molecules, which indicate an interaction of AMP with the Ag surfaces.

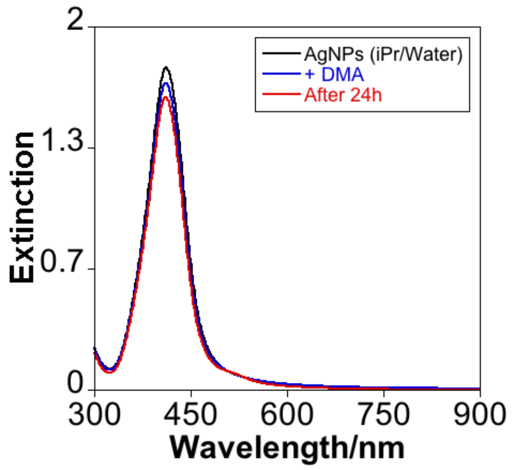

Figure S7. Extinction spectra of Ag@AMP in iPr/Water after injection of DMA and with 24h of ageing.

In the impact analysis of DMA on Ag@AMP NPs, a similar plasmon damping were noted compared with Ag@AT-SC NPs. However, prolonged contact did not produce the previously observed blue shift showed in figure S2, indicating more resistance to oxidation compared to non-functionalized AgNPs. Furthermore, plasmon damping slightly increased after extended contact with DMA, suggesting an enhanced adsorption of DMA molecules on the AgNPs with longer contact times but without oxidation.

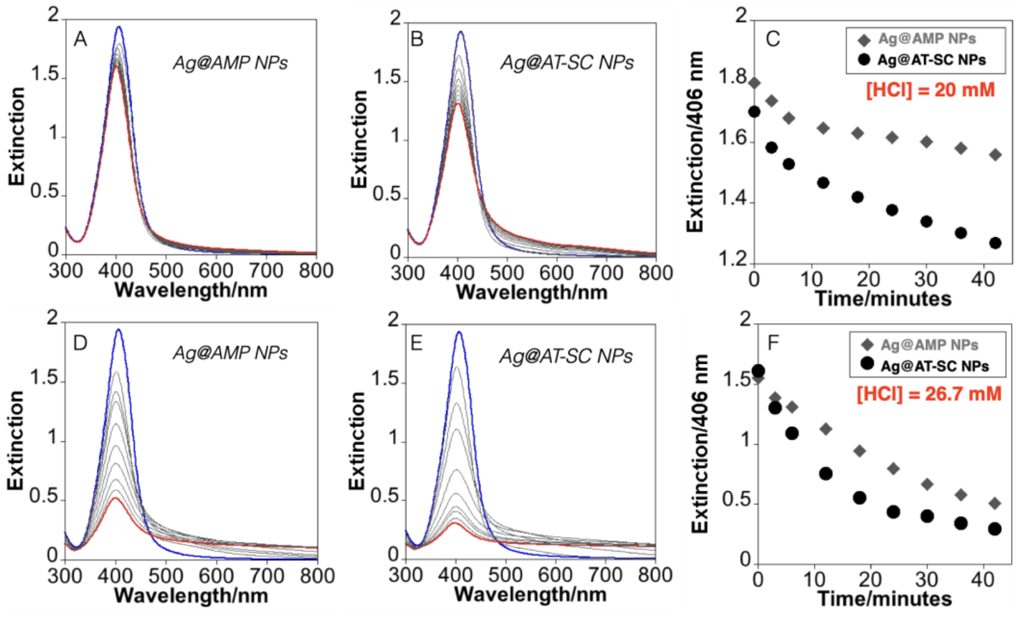

Figure S8. Spectrophotometric studies of the oxidation of Ag@AMP and Ag@AT-SC NPs in HCl at different concentration: 20 mM (A,B and C) and 26.7 mM (D, E and F). Blue spectra correspond AgNPs before addition of HCl in all cases.

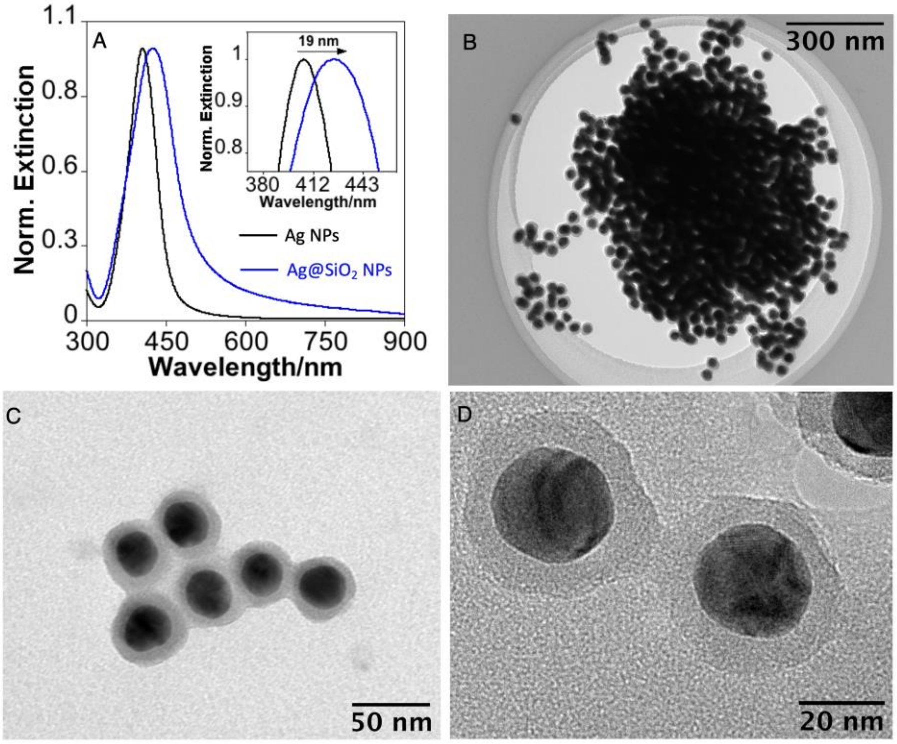

Figure S9. Normalized extinction spectra (A) and TEM images (B-D) of Ag@SiO_2 NPs with a silica thickness mean of 9.3 ± 1.8 nm obtained using 5 mL of [Ag@AMP]=5.8x10^{11} as seeds and [TEOS]= 0.37 mM.

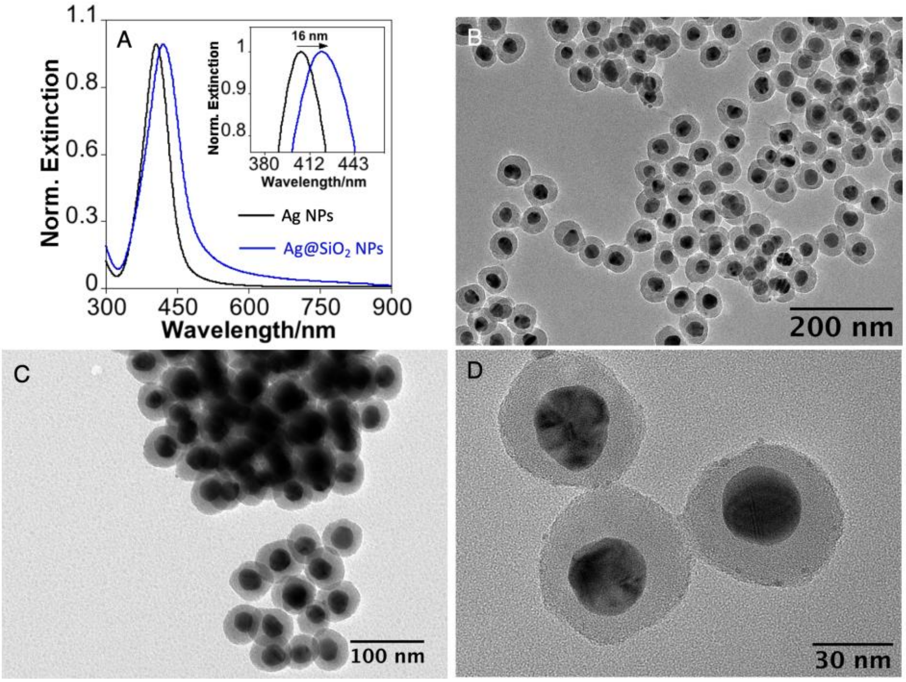

Figure S10. Normalized extinction spectra (A) and TEM images (B-D) of Ag@SiO_2 NPs with a silica thickness mean of 16.1 ± 2.8 nm obtained using 5 mL [Ag@AMP]~5.8x10^{11} as seeds and [TEOS]= 0.52 mM.

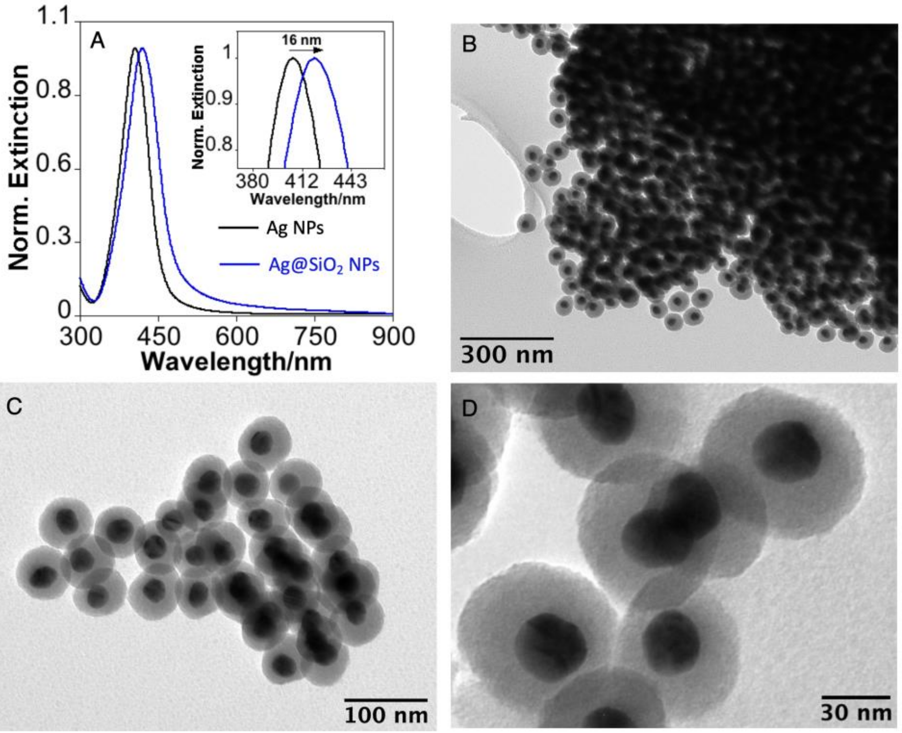

Figure S11. Normalized extinction spectra (A) and TEM images (B-D) of Ag@SiO_2 NPs with a silica thickness mean of *ca.* 20.5 ± 3.4 nm obtained using 5 mL [Ag@AMP]~5.8x10^{11} as seeds and [TEOS]= 0.67 mM

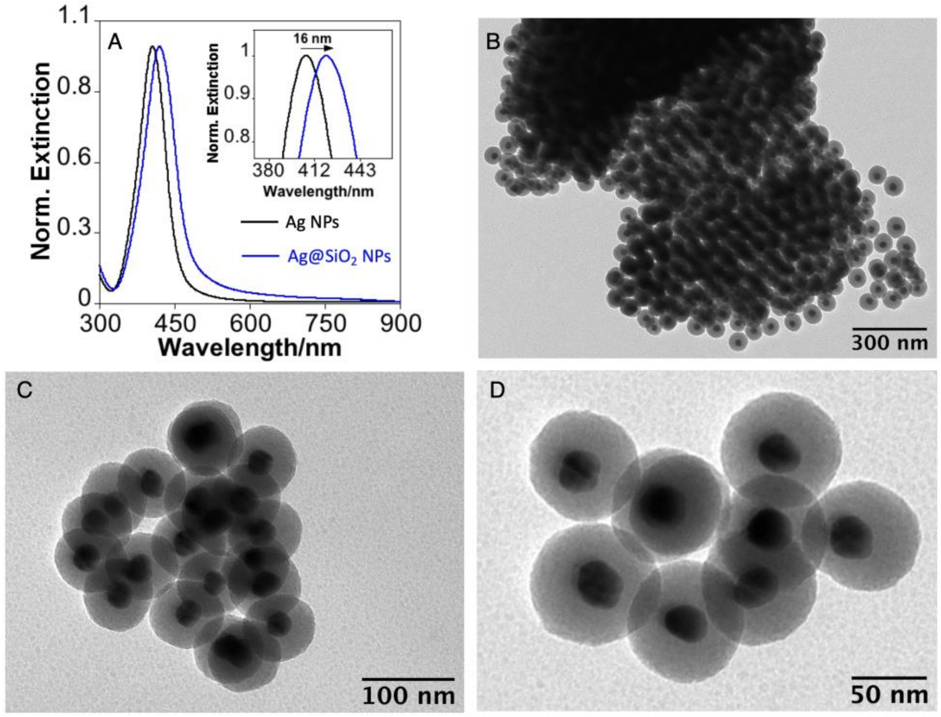

Figure S12. Normalized extinction spectra (A) and TEM images (B-D) of Ag@SiO_2 NPs with a silica thickness mean of 27.2 ± 3.8 nm obtained using 5 mL [Ag@AMP]~5.8x10^{11} as seeds and [TEOS]= 0.90 mM.

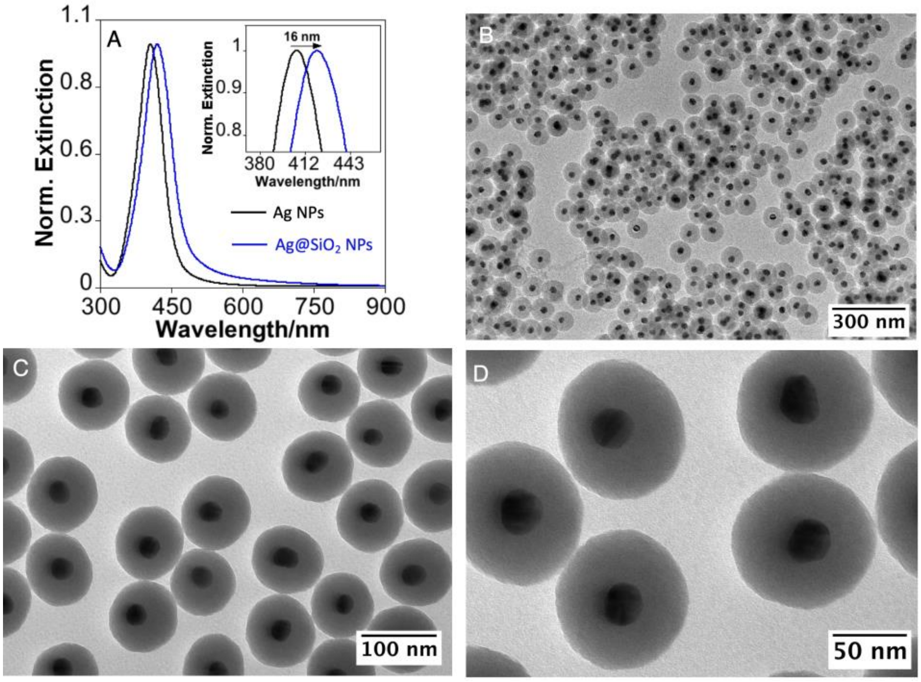

Figure S13. Normalized extinction spectra (A) and TEM images (B-D) of Ag@SiO_2 NPs with a silica thickness mean of 35 ± 5 nm obtained using 5 mL [Ag@AMP]~5.8x10^{11} as seeds and [TEOS]= 1.5 mM.

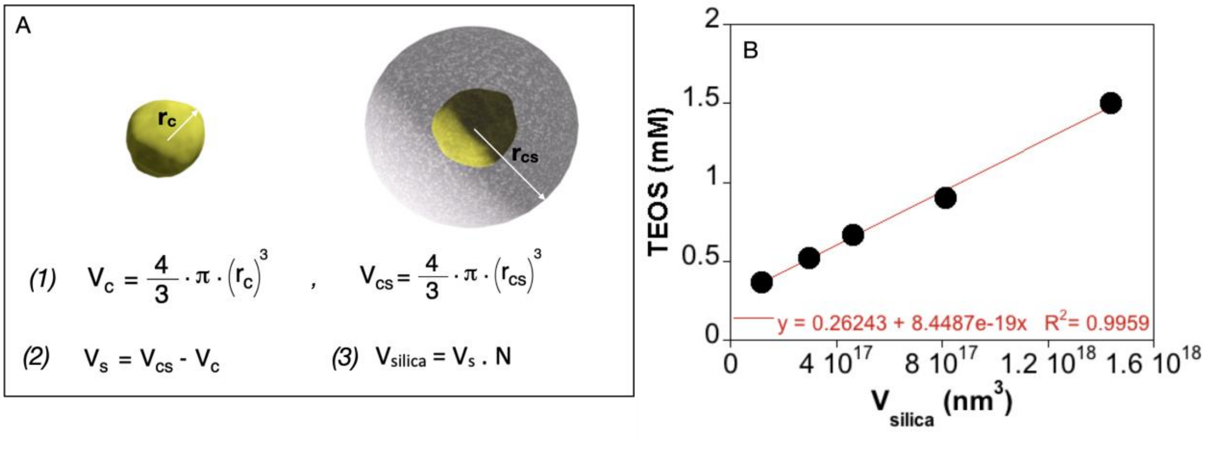

Figure S14. Illustration of the calculation approach for determining the volume of formed silica (V_{silica}) (A). The plot (B) displays varying V_{silica} values corresponding to different [TEOS] concentrations. The red equation represents data point alignment achieved using a linear regression refinement process.

The volumes of silica obtained for each [TEOS] concentration were calculated by considering the mean size of the Ag and Ag@SiO_2 NPs in each case, as determined from the TEM images, and using equations

(1) to (3), where r_c and r_{cs} correspond to the radii of the core and core@shell, respectively; V_c, V_{cs}, and V_s correspond to the volumes of the core, core@shell, and shell; V_{silica} represents the volume of silica formed, and N represents the number of nanoparticles.[3]

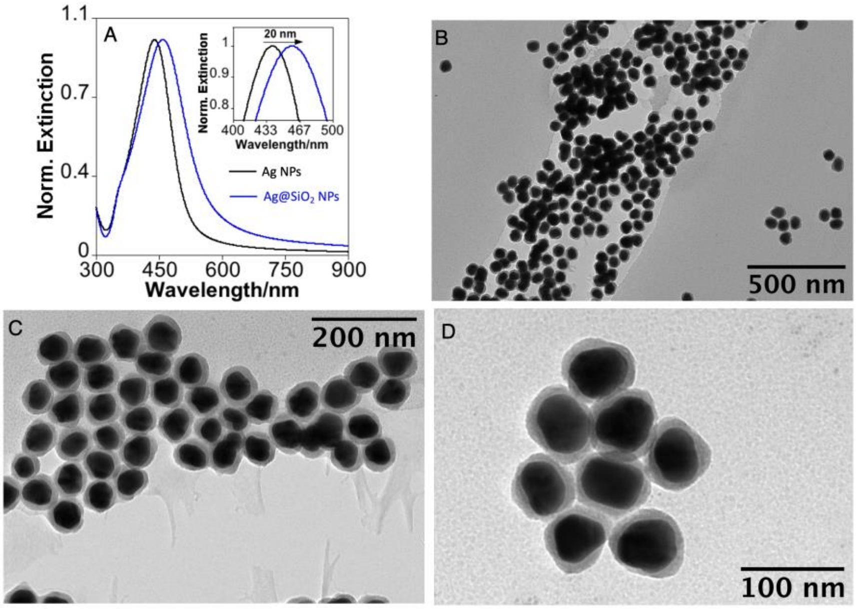

Figure S15. Normalized extinction spectra (A) and TEM images (B-D) of Ag@SiO_2 NPs with a silica thickness mean of 11.3 $\pm$ 2.0 nm obtained using 5 mL [Ag@AMP]~5.8x10^{11} with size of *ca.* 55 nm as seeds and with [TEOS]= 0.55 mM.

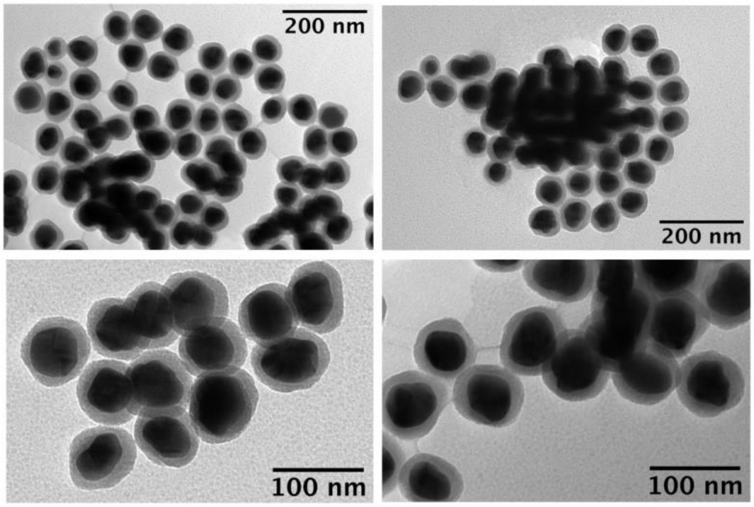

Figure S16. TEM images of Ag@SiO_2 NPs with a silica thickness mean of 13.9 $\pm$ 2.5 obtained using 5 mL [Ag@AMP]~5.8x10^{11} with size of *ca.* 55 nm as seeds and with [TEOS]= 0.7 mM.

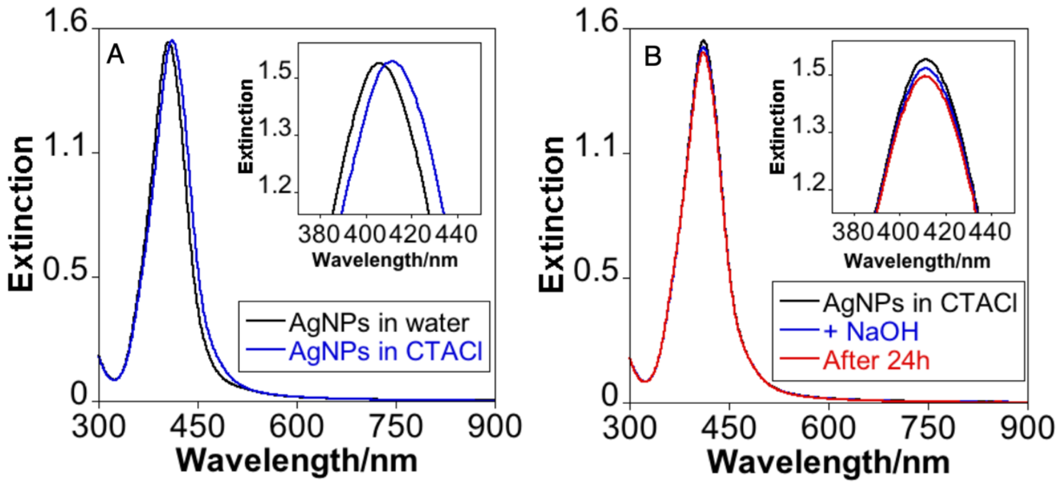

Figure S17. Comparative extinction spectra of the Ag@AMP NPs in water or in 0.8 mM of CTACl (A). Extinction spectra of AgNPs@AMP in CTACl, after adding NaOH and ageing for 24h (B).

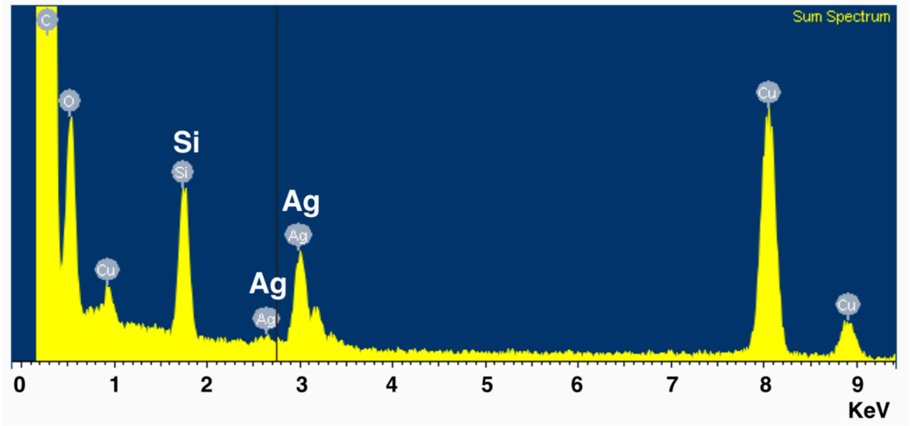

Figure S18. EDS spectrum of Ag@mSiO_2 NPs obtained for the images showed in Figure 2**E**, **F**.

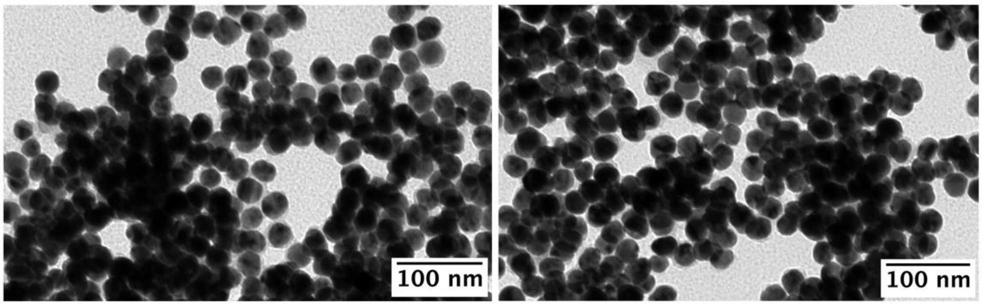

Figure S19. TEM images of Ag@mSiO_2 NPs obtained using 5 mL of [AgNPs]~5.8×10^{11} NPs/mL, [CTACl]=0.8 mM and [TEOS]=1.5 mM.

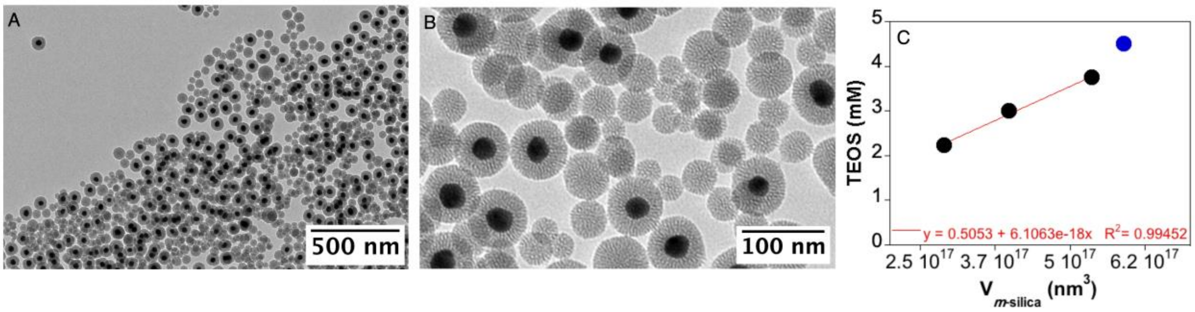

Figure S20. TEM images of Ag@mSiO$_2$ NPs (A, B) obtained using 5 mL of [AgNPs]~5.8x10^{11} NPs/mL, [CTACl]= 0.8 mM and [TEOS]=4.5 mM. The plot (C) shown V_{silica} values associated with different [TEOS] concentrations. The red equation depicts the data point alignment achieved through a linear regression refinement process. Notably, the blue point, corresponding to Ag@mSiO$_2$ NPs obtained with a TEOS concentration of 4.5 mM, deviates from the linear trend.

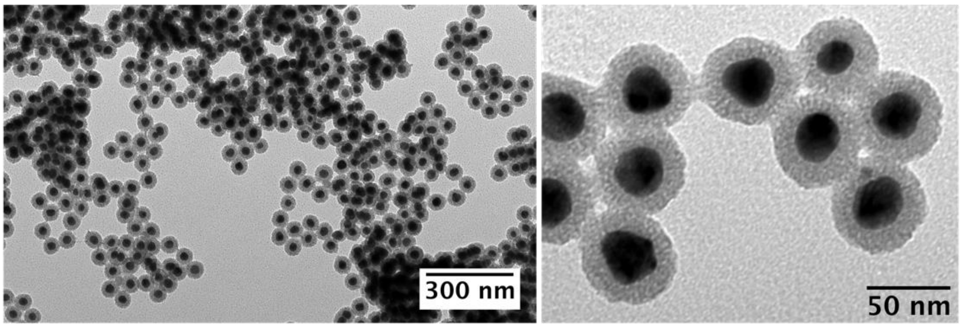

Figure S21. TEM images of Ag@mSiO$_2$ NPs obtained using 5 mL of [AgNPs]~5.8x10^{11} NPs/mL, [CTACl]=0.8 mM and [TEOS]=3 mM submitted to several centrifugation cycles using first EtOH, then MeOH and finally water as solvent.

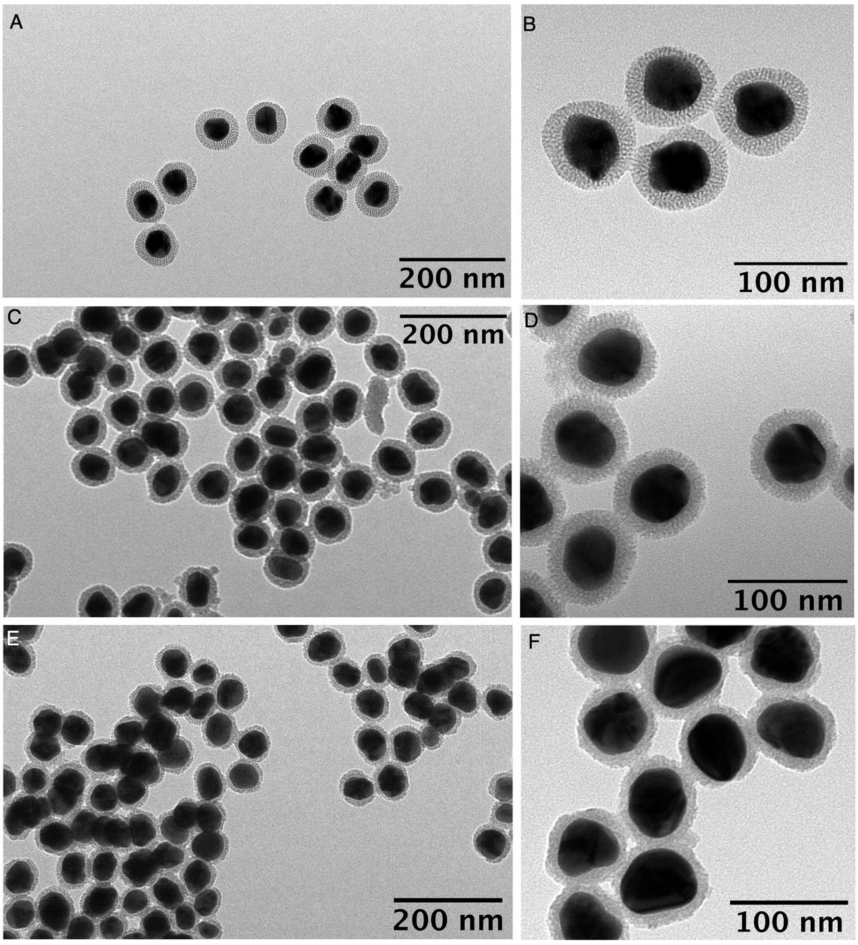

Figure S22. TEM images of Ag@mSiO_2 obtained using 5 mL of [AgNPs]~5.8x10^{11} NPs/mL of large AgNPs (*ca.* 55 nm) as seeds, [TEOS]=4 mM and with [CTAC]= 0.8 mM (A,B), 1 mM (C, D) and 1.5 mM (E, F) ranging silica thickness between 15.2 $\pm$ 1.9 nm and 11.2 $\pm$ 1.8 nm.

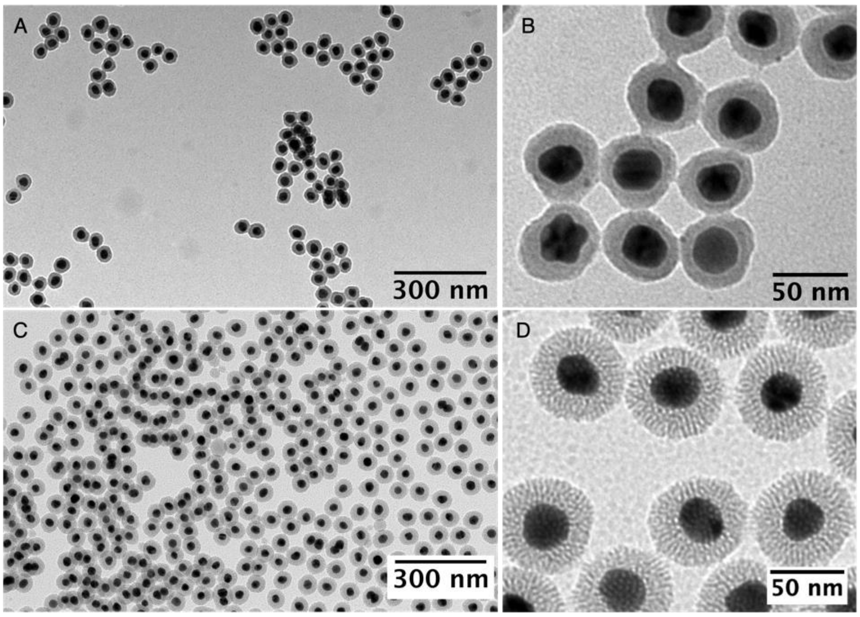

Figure S23. TEM images of Ag@SiO_2 (A, B) and Ag@mSiO_2 (C, D) obtained using 5 mL of [AgNPs]~5.8x10^{11} NPs/mL ageing 2 month in the fridge a 4°C. The aged colloidal solution was submitted to ultrasound 1 min prior to its application as seeds in silica coating.

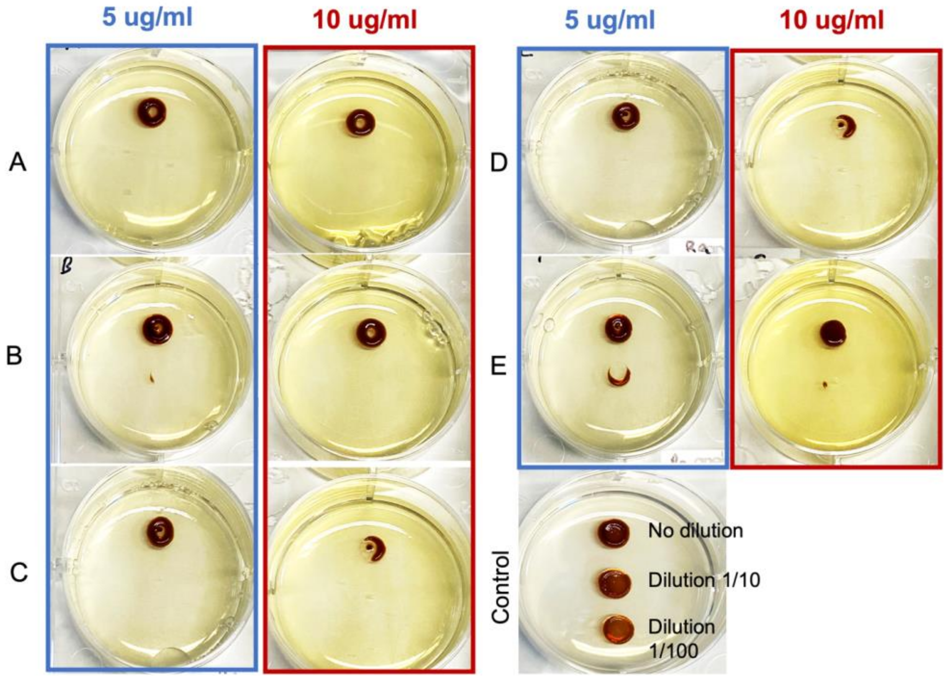

Figure S24. Petri dishes - *R. gelatinosus,* at different culture concentrations (no dilution, diluted 1/10 and diluted 1/100), growth on AgNPs (samples A-E) with different concentrations (5 and 10 µg/mL)

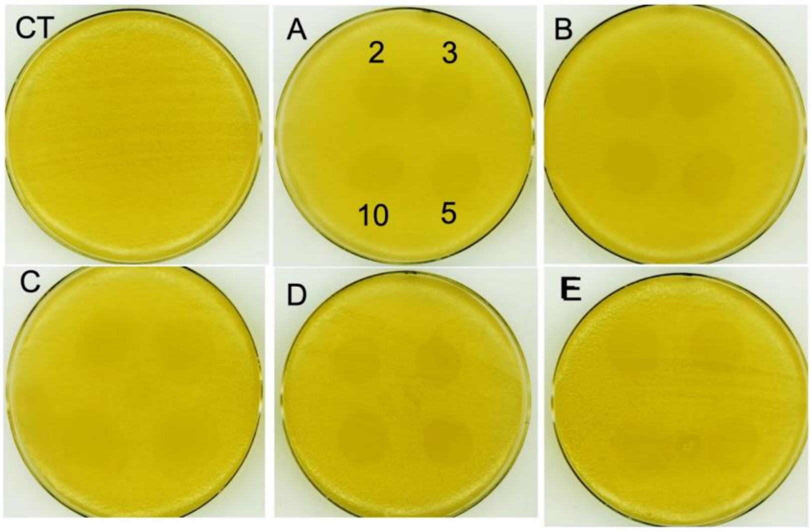

Figure S25. Growth inhibition zones on Petri dishes of *E. coli* bacteria with 2-3-5 and 10 $\mu g/mL$ of each sample of AgNPs (samples A-E)

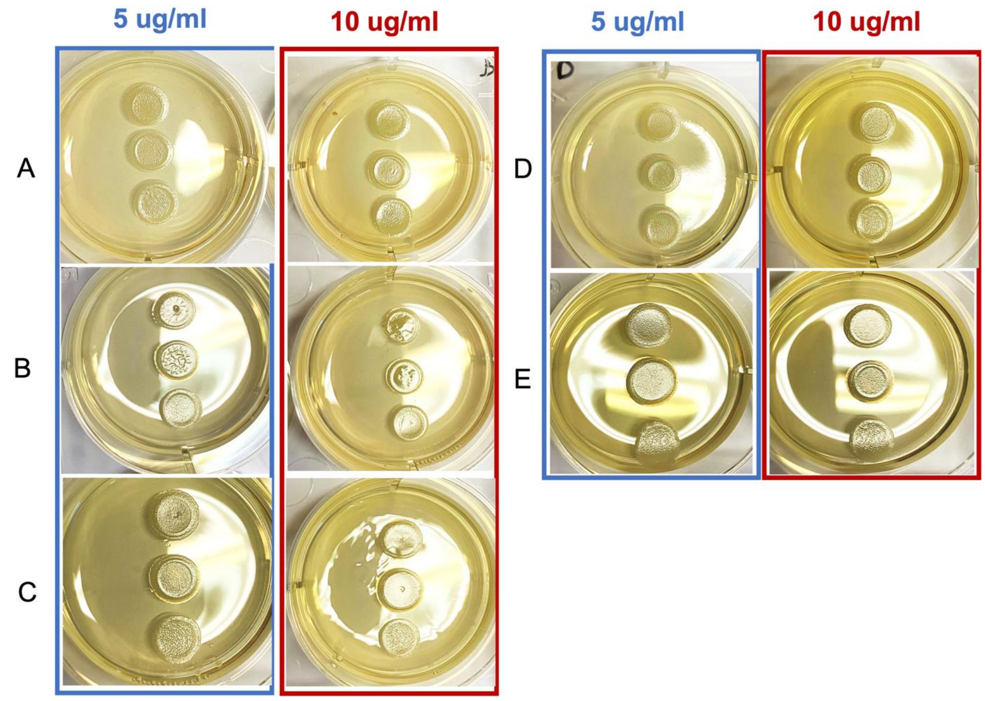

Figure S26. Petri dishes – *E. coli,* at different culture concentrations (no dilution, diluted 1/10 and diliuted 1/100), growth on AgNPs (samples A-E) with different concentration (5 and 10 μg/mL).

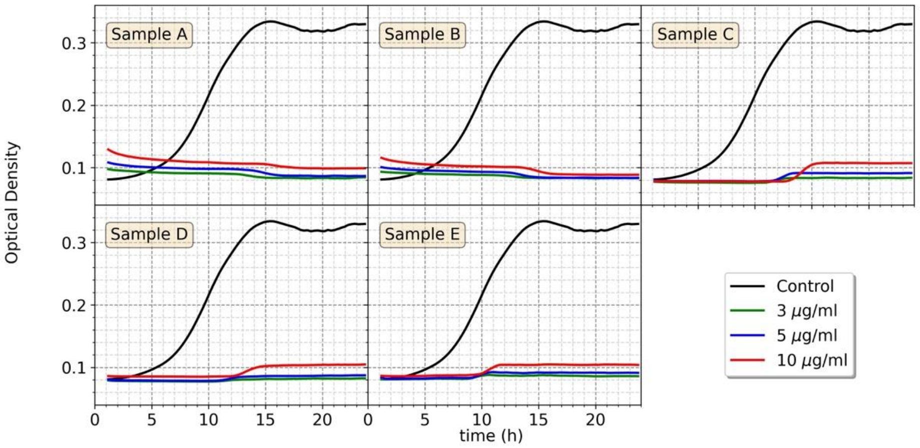

Figure S27. Growth pattern curve of *R. gelatinosus* after exposure of AgNPs (samples A-E) with concentrations 3 and 10 $\mu g/mL$.

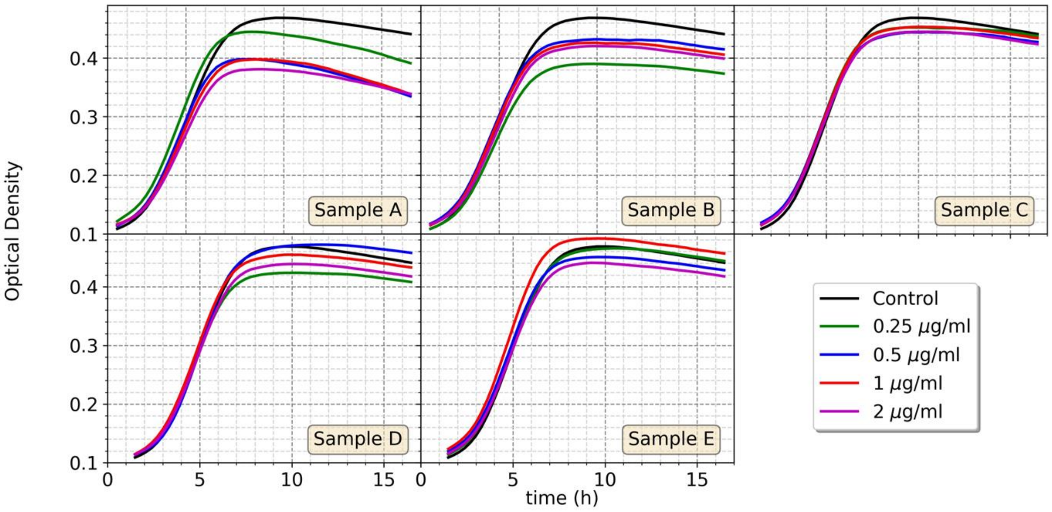

Figure S28. Growth pattern curve of *E. Coly* after exposure of AgNPs (samples A-E) with concentrations between 0.25 and 2 $\mu g/mL$.

Ag NPs	MIC (µg/mL)	Interpretation in solid medium	MIC (µg/mL)	Interpretation in solution medium
A	<2	Sensitive	≤3	Sensitive
B	<2	Sensitive	≤10	Sensitive
C	<2	Sensitive	≤10	Sensitive
D	<2	Sensitive	≤10	Sensitive
E	<2	Sensitive	≤10	Sensitive

Table S1. MIC value for *E. coli* growth pattern

References.

[1] Foerster B.; Spata V. A.; Carter E. A.; Sönnichsen C.; Link S., Plasmon damping depends on the chemical nature of the nanoparticle interface. Sci. Adv. 2019, 5, eaav0704, doi: 10.1126/sciadv.aav0.

[2] Kobayashi, Y.; Katakami, H.; Mine, E.; Nagao, D.; Konno, M.; Liz-Marzán, L. M. Silica coating of silver nanoparticles using a modified Stober method. *J. Colloid Interface Sci.* **2005**, *283*, 392–6, doi:10.1016/j.jcis.2004.08.184.

[3] H. Dong, J.D. Brennan, Rapid fabrication of core–shell silica particles using a multilayer-by-multilayer approach, Chem. Commun. 47 (2011) 1207–1209. doi:10.1039/C0CC04221H.
